# Aptamer-Based Fluorescent Biosensors for Kanamycin Detection in Food Systems: Design Strategies, Sensing Mechanisms, and Practical Applications

**DOI:** 10.3390/foods15152758

**Published:** 2026-08-05

**Authors:** Shijing Wang, Jieqiong Qiu

**Affiliations:** College of Life Sciences and Medicine, Zhejiang Sci-Tech University, Hangzhou 310018, China; 2023332864070@mails.zstu.edu.cn

**Keywords:** kanamycin, aptamer, split aptamer, label-free method, labeled method, food analysis

## Abstract

Kanamycin (KANA), a widely used aminoglycoside antibiotic in animal husbandry, is associated with residue accumulation in foods due to improper use, threatening food safety. Detecting trace KANA in complex food systems such as dairy, meat, and apicutural products remains challenging because of matrix interferences (e.g., proteins, lipids, and co-existing ions) and limitations of conventional methods, which require labor-intensive pretreatment and sophisticated instrumentation. Aptamer-based fluorescent biosensors have emerged as promising tools for rapid, sensitive KANA detection with high specificity and on-site analysis potential. DNA aptamers act as selective recognition elements that bind KANA and undergo conformational changes for efficient signal transduction. This review summarizes recent advances in fluorescent aptasensors for KANA detection, with an emphasis on food system applications. Aptamer selection strategies are outlined, highlighting split aptamers’ advantages in binding precision and structural stability. Labeled and label-free sensing modes are compared in terms of design principles, analytical performance, and suitability for complex food matrices. Attention is paid to strategies for mitigating matrix interference and improving detection reliability in real samples. Despite progress, challenges remain in sensor stability, reproducibility, and on-site deployment. Overall, aptamer-based fluorescent biosensors provide a powerful platform for rapid antibiotic residue monitoring and advance food safety-oriented sensing technologies.

## 1. Introduction

Kanamycin (KANA), an aminoglycoside antibiotic primarily produced by *Streptomyces* species, exerts potent antibacterial activity by binding to bacterial ribosomes and disrupting protein synthesis. Owing to its broad-spectrum efficacy against both Gram-positive and Gram-negative bacteria [1], KANA has been extensively applied in veterinary medicine and animal husbandry. However, its excessive or improper use has led to the accumulation of residues in animal-derived food products, raising significant concerns for food safety and public health. The ingestion of KANA-contaminated food has been associated with adverse health effects, including ototoxicity, nephrotoxicity, and respiratory complications [2]. To mitigate these risks, regulatory agencies have established maximum residue limits (MRLs), such as 150 μg/kg in milk in the European Union and 200 μg/kg in China [3].

In practical food systems, KANA residues are frequently encountered in complex matrices such as milk, meat, and honey. The intrinsic complexity of these matrices, characterized by high contents of proteins, lipids, and polysaccharides, can induce substantial analytical challenges, including nonspecific adsorption, fluorescence quenching, and signal variability [4,5]. These matrix effects severely compromise detection accuracy and reproducibility, particularly for trace-level analysis. Conventional analytical techniques, including gas chromatography (GC) [6], high-performance liquid chromatography (HPLC) [7], and liquid chromatography–mass spectrometry (LC-MS) [8], provide high sensitivity and reliable quantification of KANA residues. Nevertheless, their application in routine food monitoring is constrained by labor-intensive sample pretreatment, high instrumentation costs, and limited portability. These limitations hinder their deployment in decentralized or field-based settings, where rapid and high-throughput screening is critically required.

To address these challenges, various transduction platforms have been integrated with aptamer-based recognition for KANA detection. Electrochemical aptasensors provide advantages in miniaturization and low-cost instrumentation, but their performance can be affected by electrode fouling and nonspecific adsorption in protein-rich food matrices. Colorimetric platforms, particularly gold nanoparticle-based systems, offer simple operation and visual readout, although their sensitivity is generally lower than that of fluorescence-based approaches and often requires additional signal enhancement strategies for trace-level detection [9]. SPR- and SERS-based platforms provide valuable capabilities for monitoring molecular interactions and target-associated spectral changes, but their reliance on sophisticated instrumentation and surface engineering limits widespread application in routine food analysis [10]. Fluorescent aptasensors, in comparison, offer outstanding sensitivity, multiplexing capability, and increasing compatibility with portable readout devices such as smartphone-based detectors and paper-based microfluidic systems, making them highly attractive for rapid KANA monitoring in complex food matrices [11]. Nevertheless, fluorescence-derived analytical sensitivity should be carefully distinguished from intrinsic binding affinity, as signal amplification can substantially enhance detection performance beyond the limits dictated by aptamer–target interactions. In this context, aptamers, single-stranded nucleic acids selected via Systematic Evolution of Ligands by Exponential Enrichment (SELEX), have emerged as promising molecular recognition elements [12,13]. Compared to conventional biorecognition molecules such as antibodies and enzymes, aptamers offer advantages in chemical stability, batch-to-batch consistency, ease of synthesis, and cost efficiency [14,15]. Aptamer-based biosensors have thus been widely explored for the detection of diverse targets, including proteins [16], small molecules [17], and antibiotics [18]. In particular, fluorescence-based aptasensors have attracted considerable attention due to their high sensitivity, rapid response, and straightforward signal readout, making them well-suited for food analysis applications.

Fluorescent sensing platforms for KANA detection have evolved significantly with the development of diverse signal transduction strategies. Fluorophores used in these systems range from conventional organic dyes to structure-dependent emitters and intrinsically fluorescent nanomaterials. Structure-dependent dyes, such as SYBR Green I (SG I) for double-stranded DNA (dsDNA) [19,20] and thiazole orange (TO) and thioflavin T (ThT) for G-quadruplex (G4) structures [21,22], exhibit enhanced fluorescence upon binding to specific nucleic acid conformations. In contrast, self-emissive nanomaterials, including carbon dots (CDs) [23], upconversion nanoparticles (UCNPs) [24], and metal nanoclusters such as gold nanoclusters (AuNCs) [25], silver nanoclusters (AgNCs) [26], and copper nanoclusters (CuNCs) [27], provide stable fluorescence signals with favorable biocompatibility and resistance to environmental perturbations, making them particularly advantageous for sensing in complex food matrices. To further improve analytical sensitivity, nucleic acid amplification strategies such as rolling circle amplification (RCA) [28], strand displacement amplification (SDA) [29], hybridization chain reaction (HCR) [19], and catalytic hairpin assembly (CHA) [30] have been widely integrated, enabling signal enhancement through the formation of extended or hierarchical nucleic acid architectures.

Despite these advances, the practical application of fluorescent aptasensors in food systems remains challenging due to matrix-induced signal interference, limited sensor stability, and variability in real sample analysis. Addressing these issues requires the rational design of sensing mechanisms compatible with complex food environments, as well as strategies to improve robustness, reproducibility, and operational simplicity. To date, multiple KANA-binding aptamers have been identified, among which KANA-1 has been further engineered into split variants that reduce nonspecific folding and enhance detection performance [31]. Based on signal generation mechanisms, aptamer-based fluorescent biosensors are broadly classified into labeled and label-free systems. Labeled approaches typically employ nanomaterial-based quenchers or reporters, whereas label-free strategies rely on structure-dependent dyes or intrinsic fluorescence signals. Split aptamer designs have gained increasing attention due to their improved binding stability, simplified probe architecture, and enhanced adaptability to complex food matrices. Early comprehensive reviews by Robati et al. [32] systematically summarized both optical and electrochemical aptasensors for KANA detection, establishing the foundational framework for this research field. Subsequent reviews [10] further expanded the coverage to include emerging nanomaterial-assisted transduction strategies. However, previous reviews mainly focused on general sensing mechanisms and overall analytical performance across diverse sensing modalities and sample matrices, while providing limited discussion on several issues that are particularly important for food-oriented fluorescence aptasensors [33]. These include rational split aptamer design principles for antibiotic recognition, recent advances in fluorescence-based sensing strategies specifically developed for food safety applications, and targeted approaches for minimizing matrix interference in complex food samples. Moreover, many previous reviews discussed electrochemical, colorimetric, and fluorescent aptasensors together, whereas a dedicated analysis of fluorescence-based kanamycin-sensing platforms for practical food applications remains limited. Therefore, this review systematically addresses fluorescence aptasensors for KANA in complex food matrices, covering aptamer screening and characterization, labeled/label-free fluorescence mechanisms, split aptamer design, and anti-interference strategies. By comparatively analyzing these sensing strategies in terms of design principles, analytical performance, and applicability to real food samples, this review provides a systematic framework for the development of robust, high-performance fluorescent aptasensors for antibiotic residue monitoring in food systems.

## 2. Aptamers of KANA

**Figure 1:**
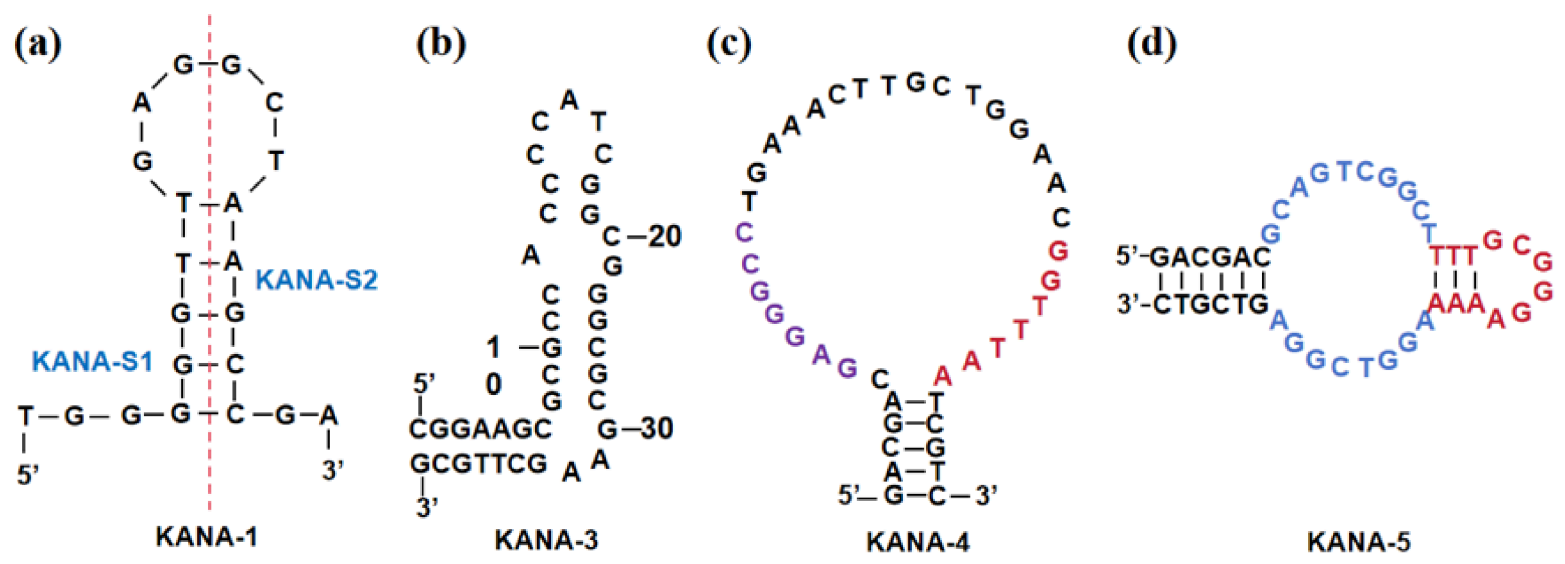
(**a**) The KANA-1 aptamer secondary structure [36], which can be subdivided into two structural domains, KANA-S1 and KANA-S2 [61]; (**b**) the KANA-3 aptamer secondary structure [40]; (**c**) the secondary structure of the KANA-4 aptamer [37]; (**d**) the KANA-5 aptamer secondary structure [41].

Aptamers are single-stranded oligonucleotides, typically comprising 15–100 nucleotides [34,35]. In 2011, Song et al. [36] developed an affinity chromatography-based SELEX strategy to isolate the first KANA aptamer, KANA-1, which exhibited specificity toward KANA (Table 1). Fluorescence analysis showed that the dissociation constant (K_d_) of this 21 nt aptamer was 78.8 nM. Notably, KANA binding led to an elevation of approximately 11 °C in the melting temperature (T_m_) of the aptamer, which was initially interpreted as strong evidence for stable complexation between the aptamer and its target (Figure 1a). However, gold-standard label-free binding assays have consistently failed to confirm the specific binding activity of KANA-1. Two independent studies using isothermal titration calorimetry (ITC)—the most reliable method for measuring solution-phase biomolecular interactions—failed to detect quantifiable specific binding between KANA-1 and free KANA, even at KANA concentrations up to 300 μM [37,38]. The originally reported 78.8 nM K_d_ was later attributed to the nonspecific adsorption of labeled aptamers to KANA-modified solid surfaces in immobilized-target assays, which are prone to overestimating binding affinity. Furthermore, it is now widely accepted that T_m_ elevation alone cannot confirm specific target binding, as it only reflects global changes in the conformational stability of the nucleic acid, regardless of whether the change is induced by specific target binding or nonspecific interactions.

This striking discrepancy has sparked extensive debate in the field. On one hand, numerous studies have developed diverse fluorescent KANA sensors using KANA-1, most of which achieved nanomolar to picomolar detection limits (Table 2) and demonstrated applicability in real matrices including milk and honey. On the other hand, gold-standard homogeneous binding assays have consistently failed to confirm its intrinsic target recognition capacity. To resolve this long-standing paradox, two plausible non-mutually exclusive explanations have been proposed. First, the early SELEX process may have enriched sequences that bound to the immobilized solid matrix or linker molecules rather than the free KANA target itself. Second, the nanomaterial-based signal amplification or nucleic acid amplification systems used in these sensors may have amplified nonspecific interaction signals, resulting in false-positive detection performance.

This inconsistency is closely associated with differences between two major characterization approaches commonly used in aptamer research [64]. One approach relies on immobilized-target assays, whereas the other evaluates solution-phase binding behavior. Immobilized assays typically measure aptamer interactions with target molecules presented on solid supports such as magnetic beads or microplates. However, surface-associated nonspecific electrostatic or hydrophobic interactions between ssDNA and solid interfaces may contribute to apparent affinity enhancement, resulting in the overestimation of the intrinsic binding capability toward free KANA [65]. In contrast, solution-phase measurements such as ITC directly evaluate the thermodynamic interaction between unbound aptamers and free targets without interference from solid supports and therefore provide more reliable information on authentic binding properties [66]. This distinction represents an important consideration in aptamer development, as candidates selected primarily based on immobilized affinity measurements may not always maintain robust recognition performance when transferred to homogeneous sensing systems or complex food matrices.

Beyond the reported variations in KANA-1 binding performance, selectivity in practical applications represents another important consideration [67]. Most KANA-1-based sensors have been evaluated against structurally unrelated antibiotics; however, their cross-reactivity toward other aminoglycosides remains insufficiently investigated. Notably, the KANA-4 aptamer exhibits considerable binding affinity toward tobramycin (TOB) [37], and the KANA-5 aptamer also shows measurable cross-binding behavior toward TOB [41]. Since the specific binding capability of KANA-1 in homogeneous solution-phase assays remains incompletely characterized, some reported sensors may partially respond to conserved structural motifs shared among aminoglycosides rather than exclusively recognizing KANA. This consideration highlights the necessity of systematic selectivity evaluation using a comprehensive panel of aminoglycoside analogs, particularly for the future translation of aptamer-based sensors into real food analysis.

Notwithstanding this unresolved core contradiction regarding KANA-1’s recognition specificity, it is still the dominant recognition probe for fluorescent KANA biosensors, and the diverse sensing architectures reported in the relevant literature have yielded valuable methodological references for the whole field of antibiotic aptasensing.

In 2012, Stoltenburg et al. [39] pioneered an innovative SELEX derivative technology, Capture-SELEX, for KANA aptamer selection, addressing the critical limitation that small organic molecules like KANA are difficult to immobilize on solid surfaces without disrupting their native structures. Unlike conventional SELEX approaches, this innovative method immobilized the oligonucleotide library rather than the target molecule via hybridization between a predefined docking sequence in the library and a complementary capture oligo conjugated to magnetic beads. After 13 rounds of iterative selection using a mixed target pool containing KANA and three other aminoglycoside antibiotics, a panel of KANA-specific aptamers was successfully obtained. Among these candidates, the Group 9 representative sequence (clone #3.19, here referred to as KANA-2.1) exhibited a K_d_ of 3.9 μM as determined by ITC, while the Group 6 representative sequence (clone #13.82, here referred to as KANA-2.2) showed a K_d_ of 5.1 μM. Building on this foundational work, Samokhvalov et al. performed post-SELEX truncation and structure-guided optimization on KANA-2.2, yielding a 42 nt variant, KANA-2b_opt, which exhibited a K_d_ of 109 ± 15 nM under near-physiological conditions. This significant affinity improvement over the parent aptamer demonstrated the power of rational truncation for aptamer performance optimization.

While Capture-SELEX solved the core problem of target immobilization without disrupting native structure, the resulting aptamers still had relatively low binding affinity under physiological conditions, prompting subsequent studies to focus on structure-guided optimization and novel screening strategies. Subsequently, in 2017, Ha et al. [40] developed an SELEX method using KANA immobilized on nitrocellulose oligosorbent (NOS)-coated 96-well plates in combination with an asymmetrically amplified ssDNA library. After nine selection rounds, three KANA-binding aptamers containing distinct 30-nucleotide randomized regions were identified. Among these candidates, KANA-3 exhibited the strongest target-binding capacity, with a K_d_ of 92.3 nM, as determined by enzyme-linked oligonucleotide assay (ELONA) (Figure 1b). This study further illustrated that KANA-3 embodied the essential sequence needed to attain significant binding affinity and specificity (Table 1), addressing the key limitation of target immobilization in conventional SELEX for small-molecule targets.

Although KANA-1 exhibited a low apparent K_d_ in immobilized-target assays, ITC evaluation estimated its intrinsic binding affinity to exceed 10 μM, highlighting the limitations of this first-generation aptamer for quantitative analytical applications under physiological conditions. To address this challenge, Zhao et al. [37] developed a capture-SELEX strategy under mildly acidic conditions (pH 6.0) to isolate KANA-4, an aptamer specific to KANA, which exhibited a K_d_ of 0.32 μM as determined by ITC (Figure 1c). Notably, the binding affinity of KANA-4 decreased progressively with increasing pH, with an affinity of approximately 2 μM at pH 7.5, a characteristic that limits its utility under physiological conditions (Table 1). This study revealed the significant impact of pH on aptamer–target binding, providing guidance for subsequent screening under physiological conditions.

Most recently, Ding et al. [41] made an important breakthrough by revisiting a previously abandoned capture-SELEX experiment conducted at pH 8.0, which had been discarded due to poor sequence enrichment. By analyzing low-abundance sequences that were overlooked in the original screening, they identified KANA-5, an aptamer with ultra-high binding affinity under physiological conditions. ITC measurements confirmed a K_d_ of 51 ± 5 nM at pH 7.5, making it the highest-affinity KANA aptamer reported to date. Nuclear magnetic resonance (NMR) spectroscopy revealed that KANA-5 underwent ligand-induced folding to form a well-defined tertiary structure with a tight binding pocket, providing a structural basis for its ultra-high affinity.

While the above studies focused on optimizing the binding affinity of full-length KANA aptamers through rational truncation and structure-guided engineering, recent advances in aptamer engineering have opened up a new direction for performance optimization: the development of split aptamers (Table 3). A key inherent limitation of long full-length aptamers is that they tend to cause steric hindrance and form unfavorable secondary structures, which can compromise binding efficiency and sensor performance [68]. In contrast, split aptamers, composed of shorter fragments, offer reduced synthesis costs, lower structural complexity, and enhanced target accessibility. In a pioneering study, Belal et al. [61] rationally split KANA-1 into two fragments, KANA-S1 and KANA-S2, and established a fluorescence detection system using Cu(I)-catalyzed azide–alkyne cycloaddition (CuAAC). Notably, this split aptamer system was developed based on the full-length KANA-1 sequence, whose intrinsic target-binding activity remains controversial as discussed above. This split aptamer design may circumvent the nonspecific binding issues associated with the full-length KANA-1 sequence, as the shorter fragments have reduced potential for nonspecific interactions with solid surfaces. However, the target-binding activity of these split fragments has not been independently validated by gold-standard solution-phase assays to date, representing a key knowledge gap in the field. Nevertheless, this approach, by enabling precise and sensitive KANA detection, laid the theoretical groundwork for the wider application of split aptamer structures in antibiotic biosensing, and inspired the subsequent development of split aptamer sensors for other small-molecule targets.

The evolution of KANA aptamers, from the controversial KANA-1 to the ITC-validated KANA-5, provides an important basis for interpreting sensor performance. A critical discrepancy emerges when comparing the data summarized in Table 1 and Table 2: many fluorescent aptasensors exhibit LOD values substantially lower than the reported K_d_ values of their corresponding aptamers. This discrepancy originates from the fundamental distinction between thermodynamic affinity and analytical sensitivity. The K_d_ value describes the equilibrium binding strength between an aptamer and its target, whereas the LOD reflects the overall signal transduction capability of the sensing system, including amplification efficiency, fluorescence enhancement, and background suppression. Nucleic acid amplification cascades can generate substantial signal enhancement from a limited number of target-binding events, thereby enabling detection at concentrations far below those expected from affinity measurements alone. In contrast, unamplified SPR and label-free SERS platforms rely more directly on binding-associated physical changes without auxiliary signal amplification, and therefore their detection capabilities are generally more closely related to intrinsic target recognition. This distinction highlights the necessity of rigorous validation for amplification-dependent fluorescent sensors, particularly when extremely low LOD values are reported, to ensure that enhanced signals originate from specific KANA recognition rather than nonspecific background amplification.

## 3. Labeled Methods

In labeled fluorescent aptasensors, fluorescence modulation is predominantly governed by fluorophore–quencher interactions. Commonly employed organic fluorophores include Cy3 [69] and Cy5 [47], which are typically paired with appropriate quenching moieties. Compared to conventional organic quenchers such as BHQ1 [70], nanomaterial-based quenchers, including gold nanoparticles (AuNPs) [43] and graphene [71], have attracted increasing interest owing to their superior quenching efficiency, broad spectral overlap, and high surface area for DNA adsorption. These features are particularly advantageous for mitigating background interference and improving signal-to-noise ratios in complex food matrices.

In parallel, nucleic acid amplification strategies, such as CHA [30], RCA [28], HCR [72], and SDA [29], have been extensively employed to further enhance detection sensitivity and specificity. Such signal amplification is crucial for the accurate determination of trace-level KANA residues in food systems, where matrix effects often compromise detection performance.

### 3.1. AuNPs as Fluorescence Quenchers

**Figure 2:**
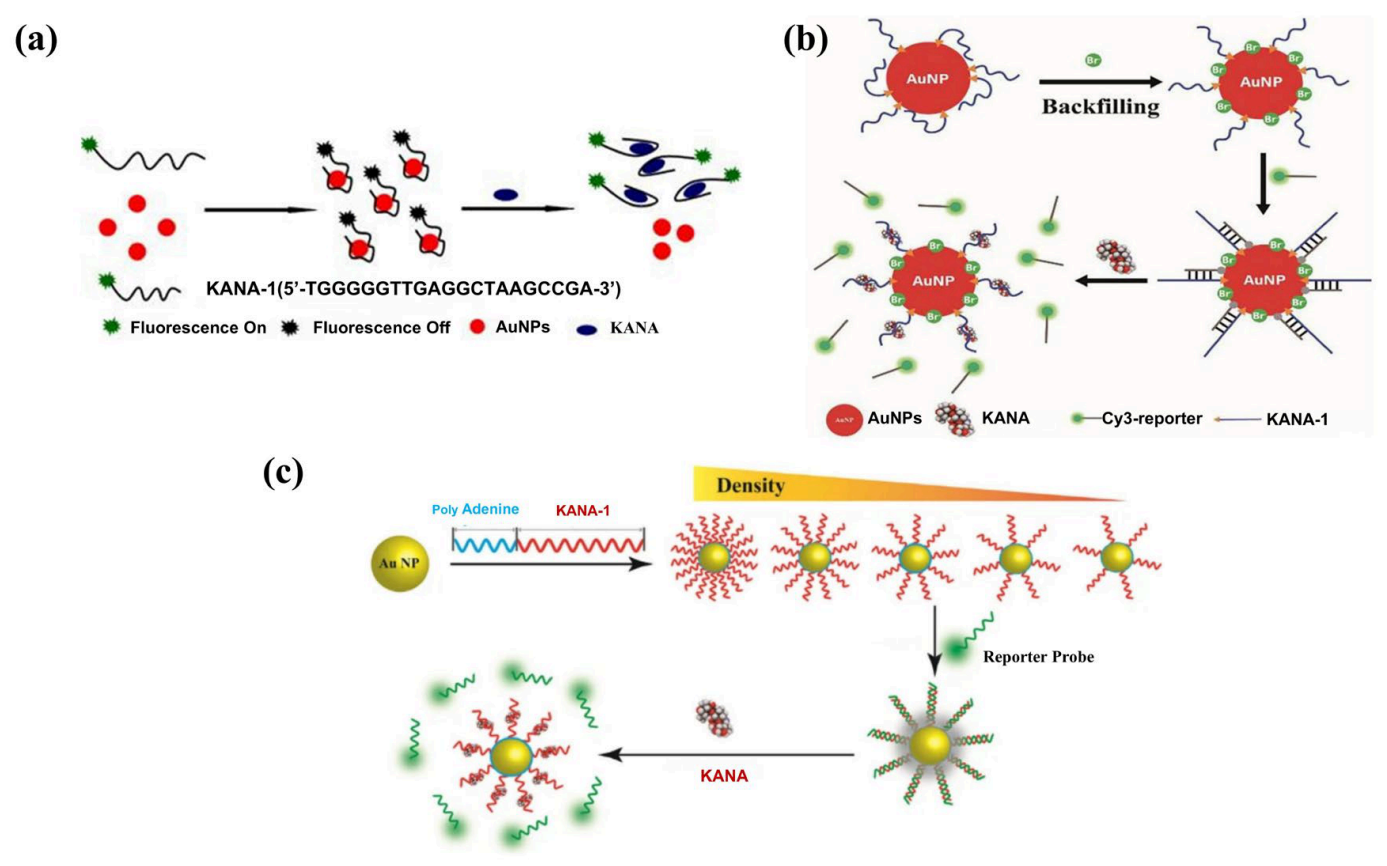
(**a**) AuNPs were employed as fluorescence quenchers and DNA nanocarriers [42]. KANA fluorescence detection using AuNPs and FAM-labeled aptamer [43]; (**b**) the application of bromide backfilling in KANA sensing for treated SNAs [44]; (**c**) utilizing AuNPs and polyA of varying lengths to modify the programmable surface density of SNAs for KANA detection [45].

AuNPs exhibit aggregation-dependent optical properties, including highly efficient fluorescence quenching, making them attractive signal modulators in aptamer-based fluorescent biosensors [9]. Owing to their excellent quenching efficiency, surface chemistry tunability, and biocompatibility, AuNPs have been widely utilized in antibiotic detection [73]. These features make AuNP-based platforms particularly suitable for rapid and sensitive KANA detection in complex food matrices. In an important study, Chen et al. [42] created an aptasensor that utilized Förster resonance energy transfer (FRET), where AuNPs functioned both as carriers for DNA and as quenchers of fluorescence for KANA (Figure 2a). An FAM-tagged aptamer was adsorbed onto AuNP surfaces without KANA present, significantly quenching fluorescence. The aptamer folded into a stable hairpin conformation upon binding to the target, a structural rearrangement that lowered its adsorption affinity toward AuNPs and restored the previously quenched fluorescence (Table 2). The practical applicability of this aptasensor was further confirmed by recovery experiments in spiked milk samples, which ranged from 99.2% to 105.1% with relative standard deviation (RSD) below 4.5%. After making improvements, Liang et al. [43] redesigned the aptamer sequence, controlled the size of gold nanoparticles, adjusted the ion environment, optimized the signal acquisition, and finally constructed a sensor that could generate detectable signals. The detection limit of this sensor was extremely low, only 0.1 pM, and the sensitivity was improved by about 3000-fold (Table 2). In this system, trace KANA binding induced aptamer desorption from the AuNP surface, leading to pronounced fluorescence recovery over an extensive linear detection range (0.1 pM–0.1 μM). Such high sensitivity was highly advantageous for detecting trace KANA residues in milk, river water, and lake water under stringent regulatory limits, as validated by recoveries of 96–103% and RSD less than 2.4% in these complex samples. FRET-based aptasensors exploit distance-dependent energy transfer between fluorophores and quenchers to achieve efficient signal modulation with high signal contrast. However, the requirement for the dual labeling of nucleic acid probes increases probe design complexity and overall assay cost.

Beyond single-stranded adsorption-based designs, Bai et al. [44] introduced spherical nucleic acid (SNA) architectures for KANA sensing (Figure 2b). In these systems, fluorophore-labeled reporter strands were quenched upon hybridization with AuNP-based SNAs, whereas target-induced aptamer binding triggered reporter dissociation and fluorescence recovery (Table 2). Further refinement using diblock oligonucleotides with polyadenine anchors enabled tunable probe configurations, with optimized constructs achieving detection limits down to 23.6 nM (Figure 2c) [45]. The structural programmability of SNAs offered enhanced robustness and adaptability, facilitating reliable KANA detection in milk with recoveries of 96.33–99.47% and RSDs of 1.2–6.9% in spiked sample analysis, while the inter-assay RSD of this SNA-based platform was 5.3%, reflecting satisfactory batch-to-batch reproducibility (Table 2).

However, despite these promising results, the practical application of AuNP-based fluorescent aptasensors in real-world protein-rich food matrices requires careful scrutiny. Wang et al. [74] systematically demonstrated that proteins in serum and milk spontaneously adsorb onto AuNP surfaces, forming a protective corona that significantly reduces the adsorption efficiency of single-stranded DNA. Importantly, the adsorbed DNA binds to the protein corona rather than directly to the AuNP surface. These findings directly challenge the signal transduction mechanism of many AuNP-based fluorescent aptasensors—where target-induced aptamer desorption from AuNPs is essential for fluorescence recovery. In the presence of a protein corona, both the initial aptamer loading and the subsequent target-dependent fluorescence response may be severely compromised, leading to unreliable detection in real samples such as raw milk or serum-containing dairy products. Consequently, while AuNP-based fluorescent aptasensors perform robustly in clean buffers and spiked samples, their direct deployment in complex unprocessed food matrices remains questionable without additional matrix-mitigation strategies.

### 3.2. Graphene/CNTs as Fluorescence Quenchers

**Figure 3:**
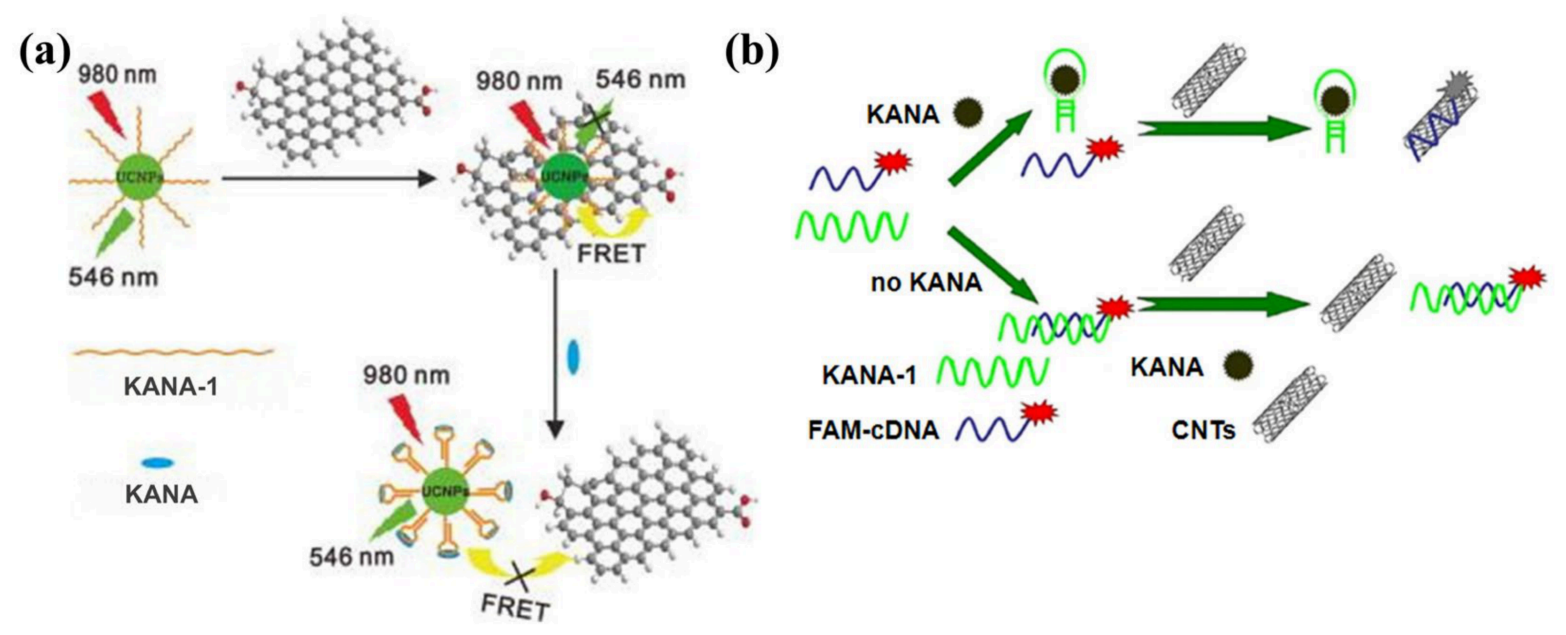
(**a**) KANA aptasensor based on FRET between UCNP-linked aptamers and graphene [24]; (**b**) utilizing CNTs as quenchers for KANA detection [46].

Graphene and carbon nanotubes (CNTs) have emerged as efficient fluorescence quenchers due to their strong π-π interactions with nucleic acids and excellent energy transfer capabilities. Their high quenching efficiency and rapid response make them well-suited for sensitive KANA detection in food safety applications. Li et al. [24] utilized upconversion fluorescence resonance energy transfer (UC-FRET) to construct a remarkably sensitive KANA aptamer sensor for trace antibiotic analysis. In this setup, UCNPs provided the donor energy, while graphene acted as the energy-accepting component (Figure 3a). Without KANA, the KANA-1 aptamer adhered to graphene through π-π stacking and assumed a random-coil structure. This adsorption event brought the UCNPs into close proximity to graphene, leading to efficient quenching of UCNP emission. When KANA was introduced, this aptamer bound specifically to the target molecule, causing its shape to change and form a hairpin structure. This change greatly reduced its ability to combine with graphene. Consequently, the increased spatial separation between UCNPs and graphene suppressed energy transfer and led to fluorescence recovery. In buffered aqueous solutions, the fluorescence signal change was proportional to the logarithm of KANA concentration, which ranged from 0.01 to 3 nM. The detection sensitivity of the whole method reached 9 pM (Table 2). This high sensitivity enabled the effective monitoring of trace KANA residues in human serum, which was critical for the clinical evaluation of antibiotic exposure and drug safety assessment.

Compared to graphene oxide (GO), CNTs exhibit more pronounced selectivity in their interactions with ssDNA and dsDNA: CNTs strongly adsorb flexible ssDNA through π-π stacking interactions, whereas their affinity for rigid dsDNA is substantially weaker [75]. Leveraging this property, Liao et al. [46] created a fluorescence-based aptasensing strategy for KANA detection using CNTs as quenchers (Figure 3b). In this system, CNTs efficiently quenched the fluorescence of an FAM-labeled KANA-1 aptamer via surface energy transfer (SET) upon adsorption. Without KANA, the labeled aptamer combined with its complementary strand to create a stable duplex, which resisted CNT adsorption and retained strong fluorescence. Upon KANA introduction, target binding induced duplex dissociation, releasing the labeled strand into solution, where it was subsequently adsorbed onto CNTs and subjected to fluorescence quenching. This sensing system exhibited excellent accuracy for KANA quantification, with an LOD of 0.4 nmol/L and a linear response range of 1.0 to 50 nmol/L (Table 2). Such selective adsorption-based sensing mechanisms are advantageous for improving detection specificity in milk, where the assay yielded recoveries of 98.5–103% with RSDs of 3.81–4.67%.

### 3.3. Signal Amplification

Nucleic acid signal amplification strategies predominantly include CHA [30], HCR [72], RCA [28], and SDA [76]. Among these approaches, CHA and HCR are emblematic enzyme-free amplification modalities, whereas RCA and SDA rely on enzymatic catalysis. By dramatically amplifying fluorescence outputs while simultaneously suppressing background interference, these strategies substantially enhance analytical sensitivity and signal robustness, which is critical for trace KANA residue detection in food matrices.

**Figure 4:**
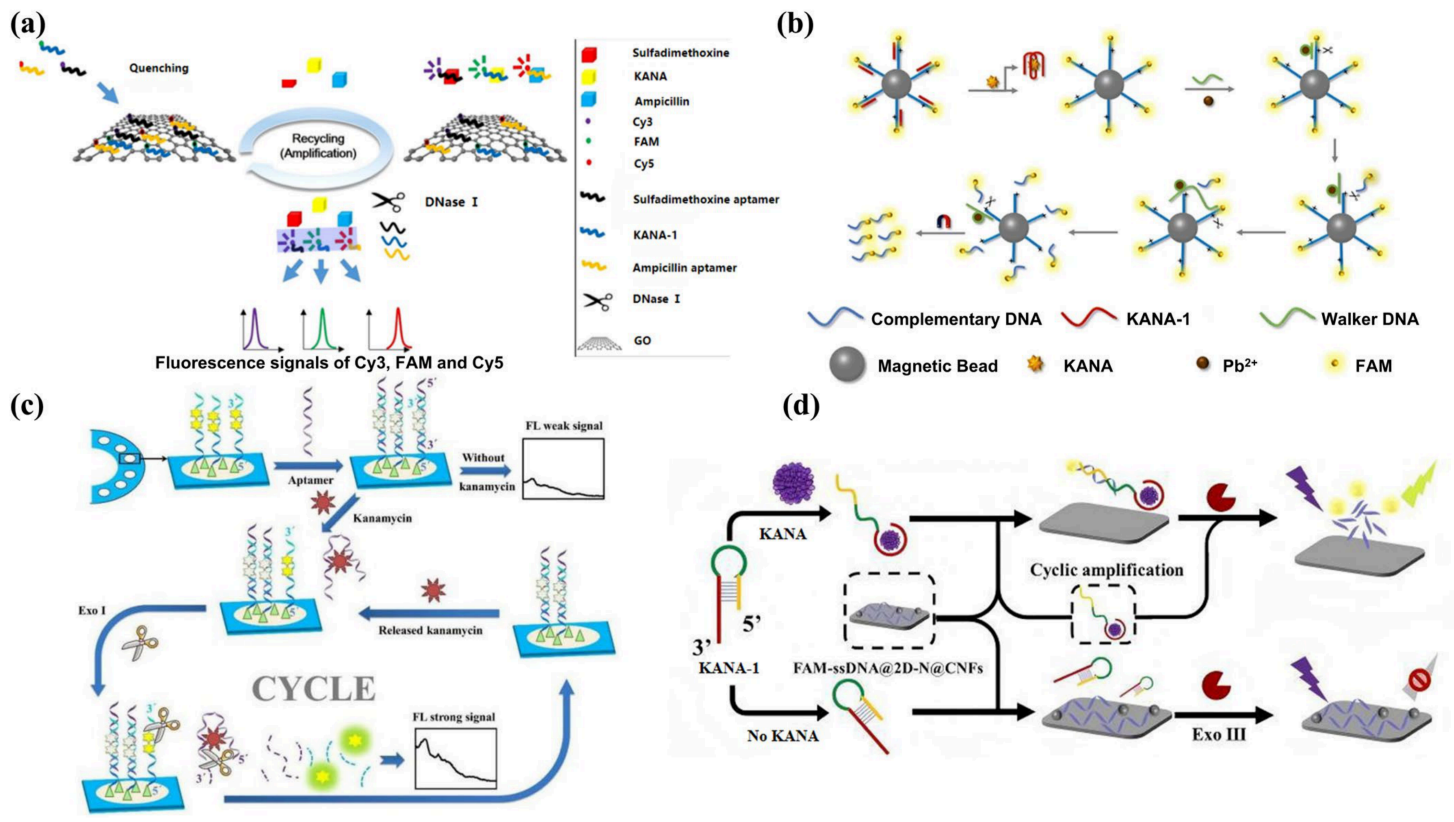
(**a**) Multiplex GO aptasensor for KANA detection via the CESA strategy [47]; (**b**) the fluorescence detection of KANA in conjunction with the amplification via DNA walkers [48]; (**c**) an ultrasensitive detection method for KANA utilizing the KANA-1 aptamer alongside a quencher-free 2-AP fluorescent probe and Exo I [49]; (**d**) sensing mechanism of the 2D-N@CNF-based sensor for KANA detection [50].

For ultrasensitive KANA detection, enzyme-assisted aptamer-based fluorescent DNA biosensors are particularly attractive, as enzymatic catalysis enables a single target–aptamer recognition event to trigger multiple reaction cycles and markedly amplify fluorescence signals. Youn et al. [47] integrated an aptamer/GO FRET system with the Cyclic Enzyme Signal Amplification (CESA) strategy driven by DNase I to develop a highly sensitive KANA aptasensor (Figure 4a). Upon KANA binding, DNase I selectively cleaved the KANA–aptamer complex, continuously releasing fluorophores and regenerating free KANA (Table 2). This cyclic amplification mechanism significantly improved detection sensitivity, enabling reliable KANA quantification in milk with satisfactory recoveries and RSD below 5%.

To further enhance amplification efficiency, Yang et al. [48] established a fluorescent aptasensor employing a DNAzyme-powered DNA walker system (Figure 4b). Initially, the KANA-1 aptamer hybridized with a complementary DNA (cDNA) immobilized on magnetic beads (MBs), forming a stable duplex. Upon KANA binding, the preferential binding of the aptamer toward KANA triggered duplex unwinding and cDNA release, which subsequently recruited walker DNA onto the MB surface. Activation of the DNAzyme domain enabled site-specific cleavage of MB-anchored, fluorophore-labeled substrates, driving autonomous walker migration through successive cleavage-displacement events. This cyclic process continuously liberated fluorescent probes, resulting in cumulative signal amplification (Table 2). Such autonomous amplification systems are particularly suitable for sensitive detection in milk with low analyte concentrations, achieving recoveries of 97.0–104.4% and RSD below 5%.

Beyond conventional fluorophores, 2-aminopurine (2-AP), which serves as a fluorescent counterpart to adenine, has been employed as an intrinsic signal reporter due to its strong fluorescence upon enzymatic release [77]. Toward the ultrasensitive detection of KANA, He et al. [49] fabricated a multipath microfluidic paper-based analytical device (μPAD), in which exonuclease I (Exo I) mediated digestion, releasing highly emissive 2-AP mononucleotides (Figure 4c). Target binding induced the disruption of the duplex architecture, enabling Exo I to simultaneously digest the 2-AP-labeled strand and the KANA-1 aptamer. This process generated abundant fluorescent mononucleotides while regenerating free KANA to initiate successive binding–hydrolysis cycles, thereby achieving efficient signal amplification. With optimal conditions in place, the assay exhibited a linear range from 10^−13^ to 10^−7^ M, while achieving an impressively minimal detection threshold of 1.26 × 10^−14^ M (Table 2). The integration of μPAD platforms highlighted the potential for portable and on-site KANA detection in food safety monitoring, with recoveries of 93.33–107.9% and RSD below 2.1% in milk and honey. Beyond paper-based microfluidic chips, miniature portable fluorometers and smartphone-assisted fluorescence imaging represent two promising strategies for field-based KANA detection. Handheld fluorometers provide quantitative fluorescence measurements with high sensitivity and reproducibility, and under optimized conditions can approach the performance of laboratory-based fluorescence instruments. However, their relatively higher cost, instrument dependence, and requirement for calibration procedures may restrict widespread deployment in resource-limited settings. Smartphone-based fluorescence imaging offers a low-cost and user-friendly alternative by integrating optical signal acquisition with image-processing algorithms [78]. This approach is particularly attractive for ratiometric fluorescence sensors or systems generating distinguishable spectral features, as internal signal normalization can partially compensate for variations in illumination and imaging conditions [79]. Nevertheless, smartphone-based platforms remain susceptible to camera-to-camera variability, ambient light interference, and differences in image-processing procedures, which may affect quantitative reproducibility. Comprehensive comparative studies evaluating handheld fluorometers and smartphone-based fluorescence readouts specifically for KANA detection in diverse food matrices remain limited and warrant further investigation [80].

In a similar manner, exonuclease III (Exo III) is an enzyme that is often used in isothermal signal amplification technology. It specifically degrades dsDNA from blunt or 3′-overhanging ends while showing no activity against ssDNA. Huang et al. [50] created a fluorescence method to detect KANA, which combined the degradation of Exo III with the quenching ability of two-dimensional N-doped carbon nanoflares (2D-N@CNFs) (Figure 4d). Without KANA, ssDNA labeled with FAM stuck to 2D-N@CNFs, and the fluorescence was greatly weakened by a process called resonance energy transfer (RET). Upon KANA binding, the formation of a KANA@KANA-1 hairpin enabled duplex hybridization with the FAM-labeled strand, thereby activating Exo III digestion and releasing fluorescent FAM mononucleotides from the quencher surface (Table 2). Such enzyme-assisted systems enhanced detection sensitivity and reliability in fish. Enzyme-assisted amplification strategies, including RCA and DNA walkers, achieve ultralow detection limits through cascade signal generation. However, they generally require multiple reaction components, prolonged incubation, and the strict optimization of enzymatic conditions.

**Figure 5:**
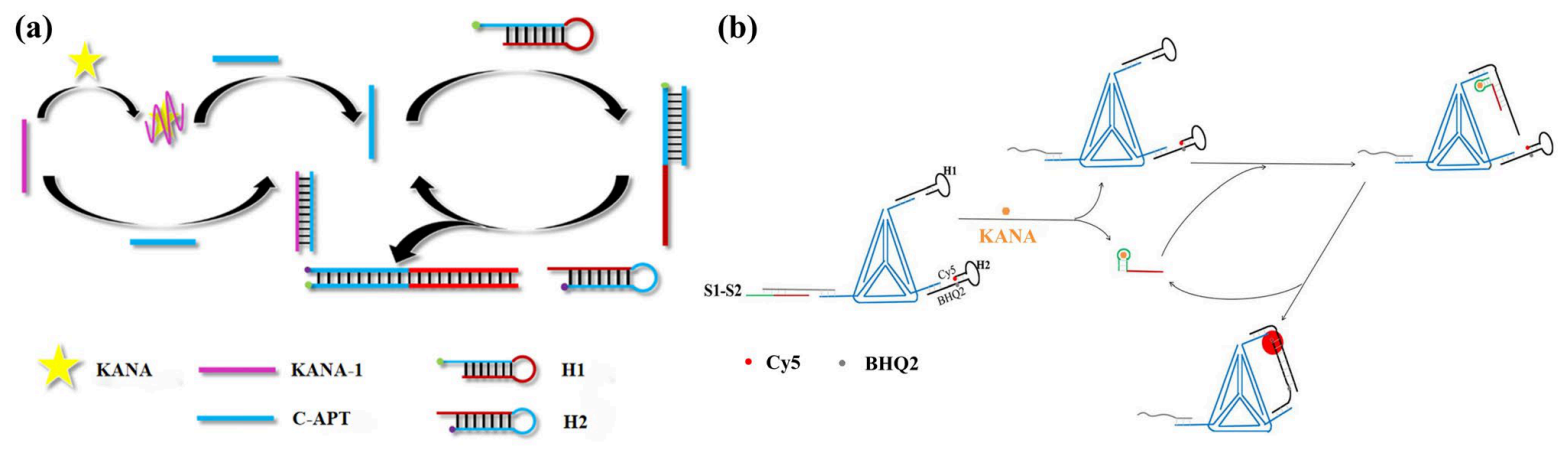
(**a**) Principle of a fluorescence quenching sensor that operates without enzymes, utilizing a competing trigger approach [51]; (**b**) L-CHA-based integrated enzyme-free fluorescent aptasensor for KANA detection [52].

Enzyme-free amplification enables simpler, more stable, and cost-effective KANA detection [81]. Bao et al. [51] designed a competitive amplification module comprising a KANA-1 aptamer, a complementary strand, and a CHA circuit formed by two hairpins (Figure 5a). In the initial state, the combination of the aptamer and its complementary chain inhibited the initiation of CHA, while the FAM fluorophore and BHQ1 quencher were spatially separated, generating intense fluorescence emission. Upon binding to its target KANA, the aptamer underwent a conformational change, released the complementary strand, and triggered the CHA amplification process. The successive activation of H1 and H2 brought FAM near BHQ1, causing a reduction in fluorescence. Continuous H1-H2 cycling enabled enzyme-free signal amplification, yielding a robust fluorescence response (Table 2). The enzyme-free nature of this system was particularly beneficial for practical food testing scenarios requiring robustness and low operational cost, as demonstrated by recoveries of 96.89–106.75% and RSDs of 1.41–2.89% in milk.

Based on the previous research, Peng et al. developed an enzyme-free fluorescent aptamer sensing system, which integrated target detection, signal conversion and local catalytic hairpin assembly (L-CHA) into a unified framework (Figure 5b). In the design, a dsDNA reactant incorporating a KANA-1 aptamer and a primer, together with two complementary hairpin probes (H1 and H2), was immobilized onto a DNA tetrahedral nanostructure. A Cy5 fluorophore and a BHQ2 quencher were immobilized on the stem of H2. Upon KANA binding, the KANA-1 aptamer embedded within the duplex underwent a target-induced conformational rearrangement, triggering duplex dissociation and exposing the primer sequence. The released primer subsequently initiated rapid H1-H2 hybridization within the confined tetrahedral framework, causing Cy5 and BHQ2 to spatially separate, which significantly enhanced the fluorescence signal. Owing to the localized L-CHA process, this integrated aptasensor enabled sensitive KANA detection within 60 min, achieving an LOD of just 0.019 ng/mL (Table 2). The integration of localized amplification and nanostructures provided a powerful strategy for rapid and sensitive KANA detection in milk, with recoveries of 96.00–108.60% and RSDs of 4.13–7.72%. Enzyme-free amplification approaches, such as HCR and CHA, reduce dependence on enzymes while maintaining substantial signal enhancement. Nevertheless, the involvement of multiple DNA components can complicate assay operation and compromise reproducibility.

The quantitative analysis of the labeled platforms summarized in Table 2 reveals that detection sensitivity is strongly influenced by both the nature of the signal transduction material and the incorporation of amplification modules. Among non-amplified nanomaterial-based sensors, AuNP-based systems exhibit a broad LOD distribution ranging from 0.0001 to 71.53 nM, indicating that their analytical performance is highly dependent on surface modification strategies, probe configuration, and assay conditions. In comparison, graphene- and CNT-based platforms generally show narrower sensitivity ranges (0.018–0.4 nM), with UCNP–graphene FRET systems achieving one of the lowest reported LODs (0.018 nM) without additional amplification.

The incorporation of nucleic acid amplification substantially improves the sensitivity of labeled platforms. Enzyme-assisted strategies based on DNase I, Exo I, or Exo III typically achieve lower detection limits through target recycling and cascade signal generation, with most reported systems reaching the sub-nanomolar range. By comparison, enzyme-free amplification approaches, including CHA, L-CHA, and HCR, provide enhanced signal amplification without enzymatic reagents, although their sensitivity improvement is often accompanied by increased probe complexity and longer reaction procedures. Notably, the superior sensitivity of amplification-assisted platforms is achieved at the expense of operational simplicity, highlighting the inherent trade-off between analytical performance and practical applicability. For non-amplified labeled systems, UCNP–graphene FRET exhibits high intrinsic sensitivity, whereas AuNP-based platforms display greater variability due to the strong dependence of signal output on probe engineering and surface interactions.

## 4. Label-Free Methods

Compared to labeled aptamer-based biosensors, label-free platforms eliminate probe labeling and purification steps, thereby reducing assay time, fabrication complexity, and batch-to-batch variability. These features make label-free systems particularly advantageous for rapid, cost-effective, and on-site detection of KANA in complex food matrices. In these systems, signal readout relies on the intrinsic responses of auxiliary reporters rather than covalently attached fluorophores, which helps minimize the structural perturbation of the aptamer and improves detection robustness in heterogeneous food environments. Label-free fluorescent DNA biosensors are mainly classified into three categories for KANA detection: intercalating dyes [53], carbon nanomaterials [57], and metal nanoclusters [26]. These platforms provide flexible signal transduction mechanisms and are well-suited for operation in complex food systems, where simplicity, stability, and resistance to matrix interference are critical for reliable analysis.

### 4.1. Intercalator Dyes

**Figure 6:**
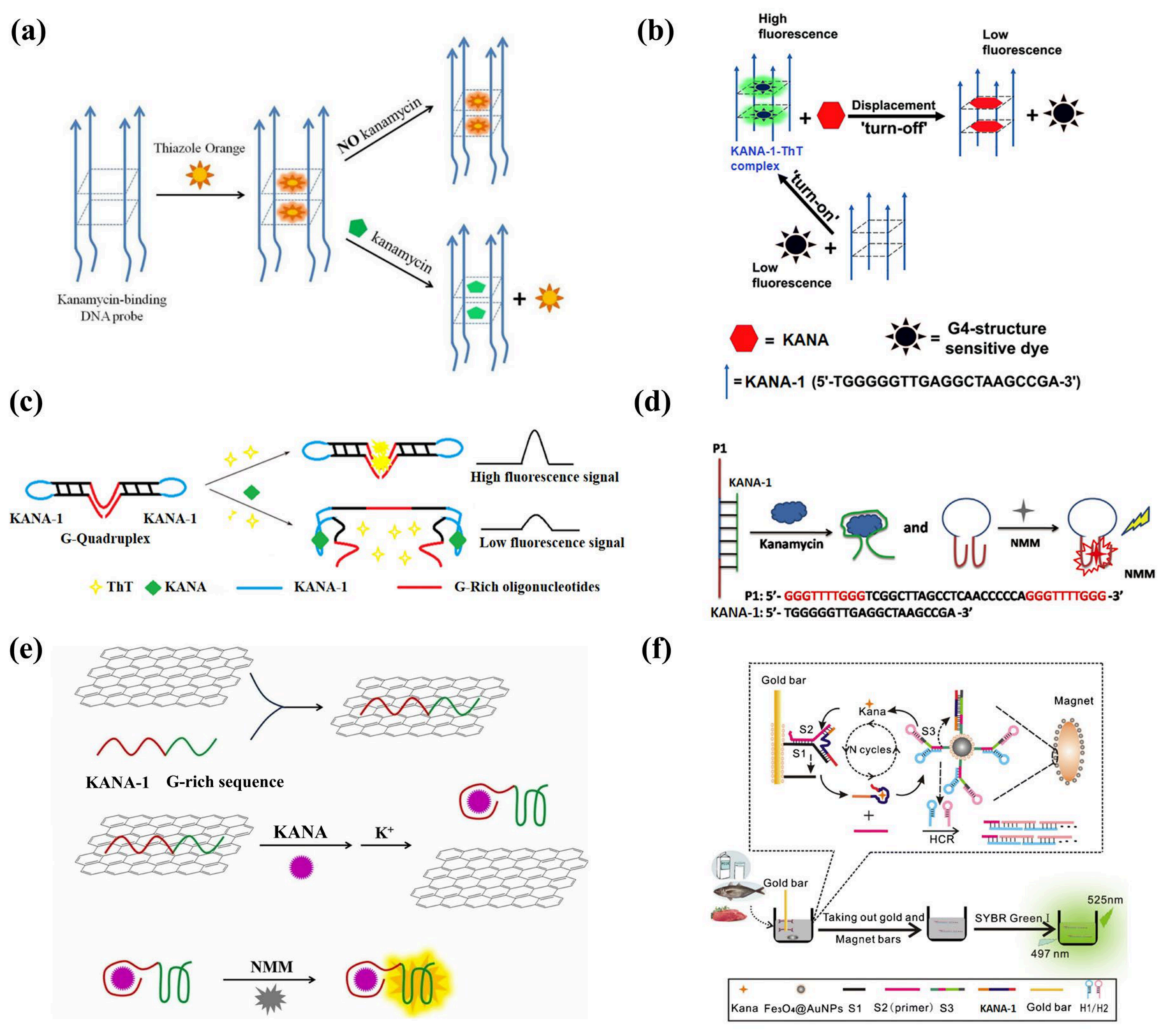
(**a**) The detection principle for KANA using a G4-DNA aptamer-based FID assay [21]; (**b**) the biosensor for KANA that operates on the principle of fluorescent displacement [53]; (**c**) an unlabeled fluorescent oligonucleotide probe that uses a G4 scaffold to recognize KANA [54]; (**d**) a novel label-free luminescent method based on DNA aptamer and NMM/G4 DNA system for KANA analysis [55]; (**e**) low-background fluorescent DNA aptasensor based on GO and NMM/G4 for KANA detection [56]; (**f**) schematic illustration of an enzyme-free fluorometric aptamer assay for KANA analysis in food samples, with target recycling triggered by dual Y-DNA structures and HCR signal amplification [19].

Structure-selective intercalating dyes, such as ThT [53] and N-methyl mesoporphyrin IX (NMM) [56], enable label-free KANA detection by transducing aptamer conformational dynamics, particularly G4 formation from G-rich sequences, into distinct fluorescence outputs. These label-free strategies are especially attractive for food analysis due to their operational simplicity and reduced susceptibility to labeling-induced variability in complex matrices. Xing et al. [21] first demonstrated that the KANA-1 aptamer could spontaneously fold into a stable tetramolecular G4 conformation, a property subsequently exploited in a fluorescent intercalator displacement assay employing TO. In this system, KANA binding displaced TO from the G4 scaffold, resulting in a concentration-dependent attenuation of fluorescence with an LOD of 59 nM (Table 2). This displacement-based sensing mechanism provided a straightforward approach for KANA screening in milk following minimal pretreatment, with recoveries of 80.1–98.0%. Substitution of TO with ThT further enhanced analytical sensitivity. Ma et al. [53] established a biosensor that utilized ThT fluorescence displacement, with the G4 motif serving as the core functional unit (Figure 6b). Upon KANA binding, the KANA-1 aptamer preferentially associated with its target, removing ThT from the G4 architecture and leading to an obvious fluorescence reduction. This platform provided a good linear correlation throughout a wide concentration window (1 nmol/L–300 μmol/L), with an LOD of just 300 pmol/L, ideal for trace KANA analysis in milk where recoveries of 94–105% were achieved with an RSD below 7.6% for four independent measurements (Table 2).

Zhu et al. [54] improved bimolecular G4-based sensing by integrating two engineered hairpin structures to enhance detection performance, thus constructing a label-free fluorescent oligonucleotide probe that employed G4 molecular beacons (Figure 6c). In this design, the KANA-1 aptamer sequence constituted the loop region of the beacon, while an adjacent G-rich segment formed a split G4 motif. Without KANA present, the beacon adopted a double-stem-loop conformation that promoted G4 assembly and ThT binding, leading to a marked fluorescence enhancement. The introduction of KANA disrupted the hairpin structure through specific KANA-1 aptamer–target interactions, leading to G4 separation and a concomitant reduction in fluorescence intensity. The lowest concentration detectable by this sensor was 0.37 nmol/L, and its response exhibited a proportional relationship over the concentration interval between 0.7 and 10 nmol/L (Table 2). The improved structural control enhanced signal specificity, which was beneficial for accurate KANA detection in milk.

In contrast to target-induced G4 dissociation, Yang et al. [55] proposed an alternative sensing paradigm based on target-induced G4 formation. They constructed a fluorescent aptasensor utilizing the NMM/G4 system (Figure 6d), wherein a G4-forming sequence (P1) was partially sequestered via hybridization to its complementary aptamer strand (KANA-1). In the absence of KANA, duplex formation between P1 and KANA-1 suppressed G4 folding, yielding negligible fluorescence upon NMM addition. Binding of KANA to KANA-1 triggered the release of P1, which subsequently self-assembled into a split G4 structure and bound NMM, producing a substantial fluorescence enhancement. This method could sensitively detect KANA with a detection range of 0.5–100 nM and an LOD of 0.5 nM (Table 2). Such “signal-on” strategies are particularly advantageous for improving detection reliability in milk with high background interference.

Further signal modulation was achieved by Cui et al. [56], who developed a ternary sensing platform integrating the KANA-1 aptamer as the recognition component, the G4/NMM combination as the fluorescent reporter, and GO as an effective quencher (Figure 6e). The composite probe, consisting of an aptamer and a G-rich sequence, was adsorbed onto the surface of GO through π-π stacking interactions, resulting in effective fluorescence quenching through FRET. Upon target introduction, the aptamer specifically bound to KANA and released the G-rich fragment. This fragment formed a G4 structure that subsequently complexed with NMM, thereby restoring fluorescence. The incorporation of GO improved quenching efficiency and signal contrast, enhancing detection performance in milk with recoveries of 98.33–101.25%, while the reproducibility of this platform was further evaluated with intra-batch RSDs of 1.50–2.17% and inter-batch RSDs of 2.51–4.76% (Table 2). G4/NMM-based label-free sensing relies on target-induced G4 folding to activate NMM fluorescence, enabling simple probe preparation and low-background signals. However, its performance is highly dependent on G-rich sequence design and ionic conditions, resulting in limited tolerance toward complex food matrices containing diverse interfering ions.

Beyond G4-selective dyes, intercalators capable of recognizing canonical dsDNA structures have also been exploited. Zhang et al. [19] developed a KANA-1 aptamer-based sensing method with SG I serving as the fluorescence indicator (Figure 6f). By integrating intercalator-based fluorescence with a dual amplification mechanism involving target recycling and HCR, the system generated abundant long-chain dsDNA upon target activation. These structures provided numerous intercalation sites for SG I, thereby enabling cascade fluorescence amplification. Owing to the synergistic effects of high dye responsiveness and dual amplification, the system could detect KANA as low as 0.45 pg/mL, realizing ultra-trace KANA detection in food systems (Table 2). Such high sensitivity is particularly valuable for ensuring compliance with stringent regulatory limits in milk, fish, and pork, where recoveries of 90.2–114.0% and RSDs of 1.13–3.25% were obtained. The above G4/intercalator displacement sensors may suffer from signal fluctuation when applied to complex dairy matrices. Naturally occurring inorganic ions and other cationic components can influence G4 folding equilibria and background dye fluorescence, introducing potential deviations independent of target-specific recognition [82,83]. Therefore, appropriate sample pretreatment and systematic matrix validation are often required to ensure reliable quantitative analysis.

### 4.2. Carbon-Based Nanomaterials

**Figure 7:**
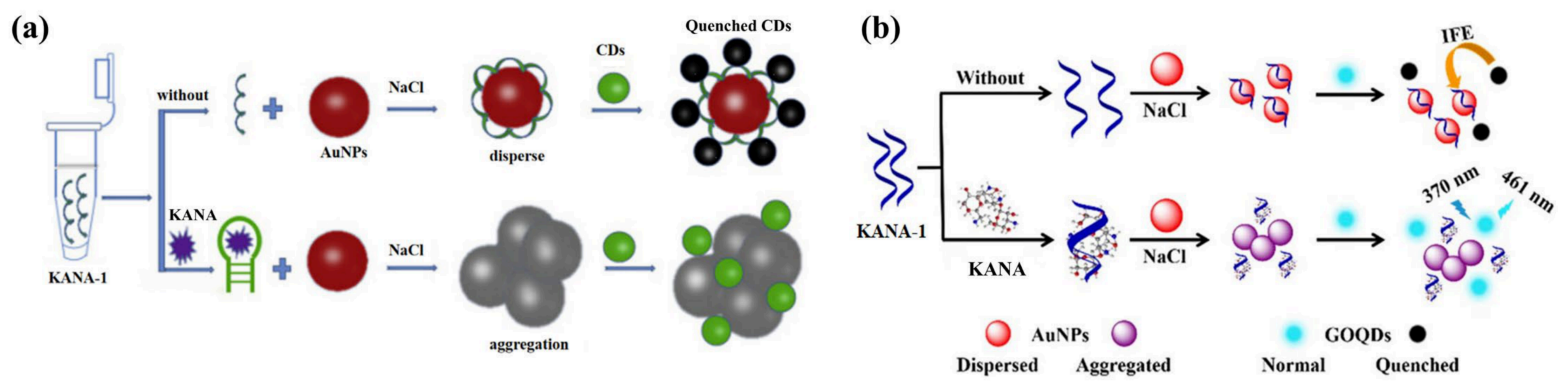
(**a**) This fluorescent aptasensor achieves quantitative detection of KANA based on the IFE [23]; (**b**) a label-free fluorescence sensing strategy utilizing the IFE between AuNPs and GOQDs [57].

Carbon nanomaterials constitute a versatile and powerful platform for biosensor construction owing to their outstanding optical performance and tunable physicochemical properties [84]. Wang et al. [23] proposed a fluorescent aptasensing platform for KANA analysis using AuNPs as absorptive quenchers to suppress CD emission via the inner filter effect (IFE). Without KANA, the adsorption of the KANA-1 aptamer onto AuNPs stabilized the nanoparticles and enabled the efficient quenching of CD fluorescence (Figure 7a). The sensor exhibited high sensitivity and could detect concentrations as low as 18 nM, meeting the demand for food safety monitoring in milk with recoveries of 90.0–98.3% and RSDs of 1.86–4.21% (Table 2).

However, the fluorescence quenching efficiency of AuNPs toward CDs was only moderate (~65%), leading to increased background signals and restricted analytical sensitivity. To overcome this limitation, Li et al. [57] introduced graphene oxide quantum dots (GOQDs) to enhance IFE efficiency in a similar label-free aptasensing system. In this coupled GOQD-AuNP sensing platform, free GOQDs act as fluorescent reporters without direct interaction with aptamer strands (Figure 7b). The IFE-based quenching mechanism originates from the spectral overlap between the absorption of dispersed AuNPs and the emission of GOQDs. In the absence of KANA, KANA-1 aptamers adsorb onto citrate-stabilized AuNP surfaces through a combination of nucleobase–gold interactions and electrostatic adsorption. This surface association stabilizes AuNPs against salt-induced aggregation and maintains efficient fluorescence quenching of GOQDs through IFE. Although the original study did not quantitatively determine aptamer surface density, immobilization efficiency, or adsorption orientation, these parameters represent important factors governing signal reproducibility and remain insufficiently standardized among nanomaterial-based aptasensors. Benefiting from this design, the optimized biosensor achieves a quenching efficiency of 72%, with an LOD of 3.6 nM and a broad linear detection range of 5–600 nM. Aptamer-mediated AuNP stabilization and target-triggered aggregation facilitated sensitive fluorescence recovery (Table 2). The improved quenching performance enhanced signal contrast, which was critical for reliable detection in milk and honey with strong background interference, achieving recoveries of 94.2–106.6% and RSD below 5.56%. IFE-based carbon nanomaterial sensors offer facile fabrication and excellent photostability. However, their signal generation primarily relies on spectral overlap rather than target-induced structural reconfiguration or amplification cascades, which generally limits sensitivity compared to amplification-assisted platforms.

### 4.3. Metal Nanoclusters

**Figure 8:**
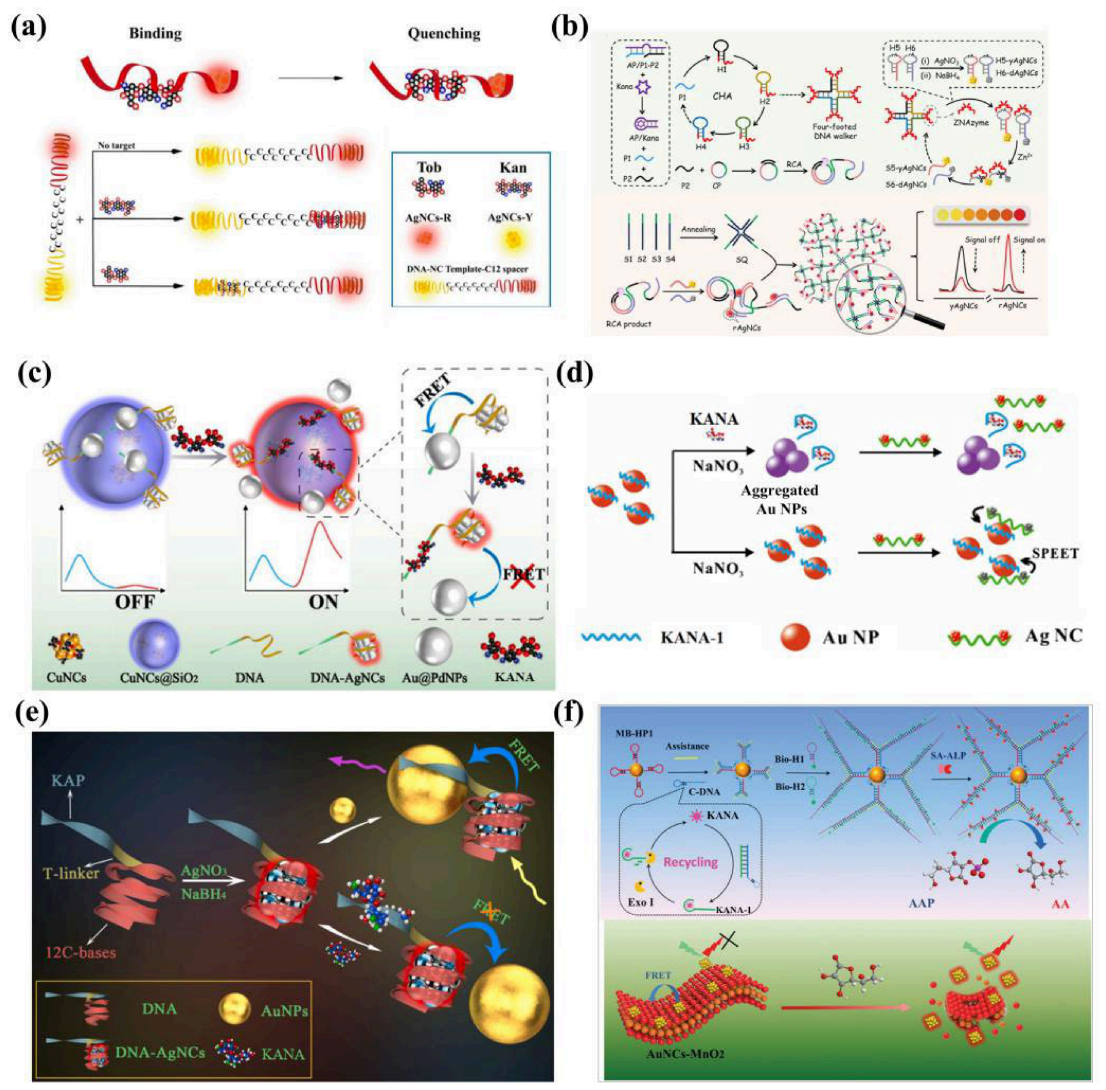
(**a**) Simultaneous fluorescence determination of KANA and TOB employing dual-emissive AgNCs stabilized by DNA [58]; (**b**) working principle of label-free ratiometric fluorescent biosensing platforms [26]; (**c**) sensing mechanism for KANA detection via the stability of CuNCs@SiO_2_ and specific binding of anti-KANA aptamers on DNA-AgNCs [27]; (**d**) schematic illustrations of KANA detection based on SPEET between AgNCs and AuNPs [59]; (**e**) schematic representation of fluorescent KANA aptasensing using DNA-AgNCs/AuNPs [60]; (**f**) schematic of an aptasensing platform constructed from AuNCs/MnO_2_ composites [25].

Metal nanoclusters have emerged as compelling alternatives owing to their ultrasmall dimensions, intense photoluminescence, and favorable biocompatibility. AgNCs are further distinguished by their unique absorption and emission characteristics. DNA-templated AgNCs, in particular, enable label-free and multiplexed sensing owing to their sequence-dependent emission behavior. Yin et al. [58] created a fluorescent biosensor based on dual-emission aptamers, which was specifically used to detect KANA and TOB simultaneously in milk (Figure 8a). Target-induced aptamer conformational changes selectively quenched the corresponding fluorescence channels, allowing quantitative dual-analyte determination with a KANA detection limit of 180 pM (Table 2). This multiplexed detection capability was particularly valuable for monitoring co-existing antibiotic residues in complex food systems such as dairy products. This dual-emissive AgNC strategy enabled the simultaneous quantification of KANA and TOB in milk matrices with satisfactory recoveries. The incorporation of a C12 alkyl spacer reduces signal interference between the two AgNC emission channels. Nevertheless, residual spectral tail overlap between fluorescent reporters remains a potential source of quantitative uncertainty, and comprehensive selectivity evaluation toward structurally related aminoglycosides is still required for broader multi-antibiotic screening applications. Moving beyond single-aptamer conformational transduction, You et al. [26] developed an extremely sensitive, label-free ratiometric fluorescent biosensor by integrating DNA nanostructure assembly with cascade signal amplification (Figure 8b). This platform was based on a cross-linked DNA network functionalized with DNA-AgNCs, assembled through KANA-1 aptamer-triggered RCA using a quadruple DNA structure as the scaffold. Concurrently, aptamer binding triggered a DNA walking reaction that released large quantities of yellow-emissive DNA-AgNCs alongside non-emissive counterparts. The close spatial organization of these AgNC species within the DNA network facilitated the in situ generation of red-emissive DNA-AgNCs, thereby establishing a robust ratiometric signal transduction mechanism (Table 2). Such a highly sensitive and ratiometric strategy was well-suited for reliable KANA quantification in milk where signal fluctuation and matrix interference are significant concerns.

Tian et al. [27] constructed a ratiometric fluorescent aptasensor relying on silica-encapsulated copper nanoclusters (CuNCs@SiO_2_) for KANA analysis (Figure 8c). In this design, CuNCs@SiO_2_ provided a stable blue emission as an internal reference, whereas DNA-templated silver nanoclusters (DNA-AgNCs) incorporating the KANA-1 aptamer functioned as the red-emissive, target-responsive signal. In the KANA-free system, fluorescence could be efficiently quenched by Au@Pd nanoparticles; upon target binding, DNA-AgNC emission was restored, producing a distinct blue-to-red color transition. This ratiometric strategy enabled KANA detection with a limit of 7.3 nM (Table 2). The built-in reference signal enhanced detection reliability, making this strategy advantageous for accurate analysis in milk, honey and beef with recoveries of 95.8–104.9% and RSD below 3%.

Combinations of AgNCs and AuNPs have also been extensively explored owing to their complementary optical properties. Ye et al. [59] developed a turn-on sensing platform via surface plasmon-enhanced energy transfer (SPEET) between AgNCs and AuNPs, which was further functionalized by surface plasmon resonance (SPR) (Figure 8d). KANA-induced aptamer recognition triggered AuNP aggregation, suppressed SPEET, and restored fluorescence (Table 2). This plasmon-enhanced sensing strategy provided a sensitive platform that could be adapted for the rapid screening of antibiotic residues in milk, with recoveries of 90.2–95.4% within 30 min. By contrast, Wang et al. [60] constructed an FRET-driven aptamer-based sensor for KANA detection in milk (Figure 8e), in which KANA binding to the KANA-1 aptamer weakened DNA-AuNP interactions, increased donor–acceptor separation, and recovered DNA-AgNC fluorescence, resulting in a detection limit of 22.6 nM (Table 2). Its successful application in milk highlighted its practical potential for routine food safety monitoring, with recoveries of 107.50–117.69% and RSDs of 1.21–4.77%.

AuNCs have similarly garnered heightened interest as fluorescent probes on account of their low cytotoxicity, outstanding water solubility, and excellent biocompatibility [85]. Zeng et al. [25] developed a dual-mode KANA biosensor by integrating AuNCs with MnO_2_ nanosheets (Figure 8f). Target detection activated a target-driven cyclic amplification system, which combined three-way junction hybridization chain reaction (3WJ-HCR) with magnetic nanoparticles, and eventually led to the accumulation of alkaline phosphatase (ALP). Subsequent enzymatic hydrolysis of ascorbyl-2-phosphate by ALP generated ascorbic acid (AA), which etched the MnO_2_ nanosheets and released AuNCs, thereby restoring fluorescence. This platform measured the fluorescence level between 0.002 and 5 nM, and its detection limit reached 1.2 pM. It enabled naked-eye readout via a brown-to-white color change (cut-off: 50 pM), albeit at the cost of a relatively long assay time (Table 2). Such dual-mode sensing with visual readout offered promising potential for rapid and on-site screening of KANA residues in milk. Metal nanocluster platforms possess unique photophysical properties, and ratiometric designs can partially compensate for matrix-induced signal drift through self-calibration. However, the complex nucleation process may introduce batch-to-batch variability, and the underlying formation mechanisms remain incompletely understood.

Quantitative comparison of label-free sensing platforms demonstrates distinct performance characteristics among different fluorescence transduction mechanisms. G4/intercalator dye systems exhibit a relatively broad sensitivity range (0.3–59 nM), where the analytical performance is largely determined by dye properties, G4 folding topology, and environmental ionic conditions. Within this category, ThT-based displacement assays generally show lower reported detection limits than TO-based systems, whereas NMM-based designs provide stronger fluorescence contrast owing to structure-specific G4 recognition. However, the dependence of G4 formation on sequence composition and surrounding ions remains a major limitation for applications in complex food matrices.

Carbon nanomaterial-based inner filter effect (IFE) platforms, including carbon dots and graphene oxide quantum dots, exhibit moderate sensitivity (3.6–18 nM) but provide advantages in terms of facile preparation, photostability, and material accessibility. Their relatively moderate sensitivity is mainly attributed to the absence of intrinsic signal amplification or target-induced structural reconfiguration.

Among label-free architectures, DNA-templated metal nanocluster systems, particularly AgNC-based sensors, demonstrate superior sensitivity, with reported LODs ranging from 1.2 × 10^−3^ to 22.6 nM. The integration of amplification cascades, such as RCA or DNA walker strategies, further improves detection capability into the femtomolar range (down to 1.35 × 10^−5^ nM). In addition, ratiometric nanocluster designs provide self-calibration capability, which is particularly valuable for mitigating matrix-induced signal fluctuations in food samples. Nevertheless, the complex nucleation process of metal nanoclusters may introduce batch-to-batch variability and remains a key challenge for large-scale practical implementation.

While label-free platforms offer distinct advantages in operational simplicity and reduced labeling-related variability, their practical performance in real food analysis remains heavily influenced by sample matrix complexity. The diversity of pretreatment protocols adopted across different studies—ranging from simple filtration and dilution to multi-step solid-phase extraction and magnetic separation—reflects the matrix-dependent nature of sample preparation. A systematic comparison of these commonly used strategies reveals distinct advantages and limitations. Simple dilution is operationally rapid and convenient, but it risks reducing trace KANA concentrations below detectable levels in highly complex matrices [86]. Protein precipitation with trichloroacetic acid effectively removes macromolecular interferents such as proteins and lipids [87]. However, this approach may cause partial analyte loss and requires additional separation steps. Solid-phase extraction offers efficient purification and enrichment, yet it demands more complicated procedures and careful optimization [88]. Magnetic separation enables simultaneous impurity removal and target concentration at the cost of higher reagent consumption and longer processing time [89]. Importantly, the choice of pretreatment strategy directly impacts reproducibility and accuracy through target recovery efficiency, nonspecific adsorption, and potential signal interference. As summarized in Table 2, no universal protocol fits all food matrix types. The selection of appropriate pretreatment should instead be determined by the specific sensing mechanism, target concentration range, and practical application requirements. These considerations are essential for translating laboratory-developed label-free aptasensors into reliable food safety monitoring tools.

## 5. Application of Split Aptamers

Long aptamer sequences are prone to forming nonspecific secondary structures, which can compromise target accessibility and binding fidelity [90]. Such structural instability may lead to increased background signals and reduced detection accuracy, particularly in complex food matrices where nonspecific interactions are prevalent. By contrast, splitting aptamers into shorter fragments effectively mitigates undesired folding, enhances conformational stability and binding specificity, and reduces synthesis cost while improving production efficiency. Importantly, these features contribute to improved signal reliability and reduced matrix interference in food sample analysis. As a result, split aptamer strategies have gained increasing attention as advantageous alternatives to full-length aptamer designs for sensitive and accurate KANA detection in food systems. Despite growing interest in split aptamer biosensors, rational fragment truncation remains challenging because universally standardized design workflows are still lacking. For split-site selection, commonly adopted strategies favor exposed flexible regions, such as loops or stem–loop junctions, distant from the core target-binding pocket to preserve target recognition elements and essential tertiary interactions. Although core-splitting approaches have been reported for several individual aptamers, these strategies remain highly sequence-dependent and cannot yet be generalized [31,68]. Fragmentation also reshapes the thermodynamic landscape of target recognition. The bimolecular assembly process introduces an additional entropic penalty and generally decreases intrinsic affinity compared to full-length aptamers. However, target-induced fragment reassembly can reduce nonspecific structural transitions and enhance recognition specificity. Structurally, aptamers containing discrete and independently foldable.

To minimize experimental trial-and-error, computational approaches have increasingly been integrated into split aptamer design. Secondary structure prediction tools (e.g., Mfold, RNAstructure, and NUPACK) assist in identifying accessible cleavage regions and evaluating fragment complementarity, while molecular docking and molecular dynamics simulations provide insights into target-induced conformational rearrangement and fragment stability [91]. Nevertheless, current split aptamer systems still face several challenges, including limited validated split candidates for small-molecule targets, insufficient structural information for rational truncation, affinity loss caused by fragment dissociation, and difficulties in optimizing fragment concentration, assembly kinetics, and matrix compatibility. These structural, thermodynamic, and computational principles provide a framework for understanding and designing KANA split aptamer sensing strategies.

**Figure 9:**
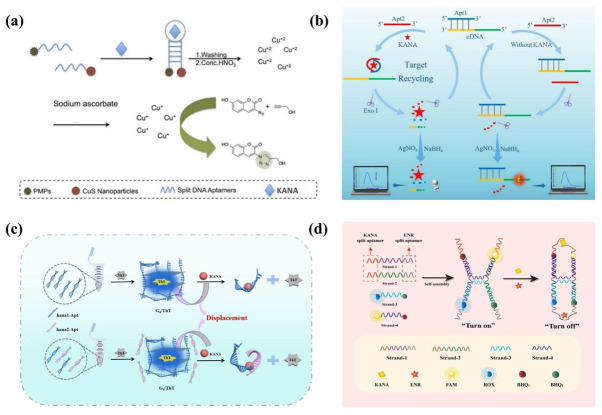
(**a**) KANA detection principle of split aptasensor based on CuAAC click reaction [61]; (**b**) fluorescent DNA biosensor with DNA-AgNCs combined with Exo I-mediated cyclic amplification for KANA detection [62]; (**c**) principle of sensing via fluorescence displacement reliant on the recognition of G4 for detecting KANA [22]; (**d**) simultaneous sensing of ENR and KANA via bifunctional DNA tweezers [63].

Belal et al. [61] first exploited a split KANA-1 aptamer, divided into KANA-S1 and KANA-S2 (Table 3), to construct a fluorescence-based detection system via CuAAC (Figure 9a). Within this sensing platform, the KANA-S1 fragment was functionalized with a 3′-terminal amine and covalently conjugated to carboxyl-modified CuS nanoparticles bearing azido groups. Meanwhile, biotin was attached to the 5′ end of KANA-S2, enabling it to be anchored onto streptavidin-coated magnetic beads through biotin–streptavidin binding. Additionally, an alkyne group was incorporated as a reactive handle. In the absence of KANA, the two aptamer fragments remained spatially separated, preventing the azide and alkyne groups from coming into proximity and thus inhibiting the CuAAC reaction. Upon target addition, KANA-S1 and KANA-S2 assembled into a target-mediated complex, bringing the reactive groups into proximity and enabling magnetic separation of the complex. Upon the addition of sodium ascorbate, Cu^2+^ was reduced in situ to catalytically active Cu^+^, which subsequently mediated the CuAAC reaction between 3-azido and 7-hydroxycoumarin and propargyl alcohol, producing a highly fluorescent 1,4-disubstituted-1,2,3-triazole product. The fluorescence intensity of the resulting triazole was directly proportional to the concentration of KANA in the sample (Table 2). This strategy demonstrated the feasibility of integrating click chemistry with aptamer recognition for selective KANA detection, offering potential for application in human serum following appropriate pretreatment. The long-term reproducibility of this split aptamer-based assay was confirmed by day-to-day RSDs of 3.5–3.9% over seven days and an overall RSD of 4.1% over eight weeks using independently prepared reagent batches.

To further enhance sensitivity, signal amplification strategies have been integrated with split aptamers. Xu et al. [62] established a KANA detection platform employing split aptamers that integrated DNA-AgNCs as fluorescent reporters, utilizing Exo I-assisted catalytic signal amplification (Figure 9b). The recognition and transduction module comprised split aptamers KANA-S1 and KANA-S2 together with a cDNA of KANA-S1. KANA-S1 fully hybridized with the 3′-terminal fragment of cDNA to form a duplex, while the 5′-terminal fragment of cDNA served as a template for the synthesis of DNA-AgNCs. Following treatment with AgNO_3_ and NaBH_4_, DNA-AgNCs with excellent stability and strong fluorescence were obtained. Upon the addition of KANA, cooperative interactions between the target and both KANA-S1 and KANA-S2 triggered the release of KANA-S1 from the pre-formed duplex. The released strands, together with cDNA, were subsequently digested by Exo I, leading to an obvious reduction in DNA-AgNC fluorescence. In the absence of KANA, Exo I selectively digested KANA-S2 while leaving the KANA-S1/cDNA duplex intact, thereby preserving fluorescence. Exo I-assisted amplification enhanced the fluorescence response by 48.98%, affording an LOD of 1.07 nM (Table 2). Notably, the assay was completed within 45 min, representing a substantial improvement in response time compared to the CuAAC-based platform. Such rapid and sensitive performance was highly desirable for the detection of trace KANA residues in oyster meat, seawater, lake water and milk, matrices that demand high sensitivity and rapid analysis.

Beyond simple binding-induced assembly, split aptamers can also modulate pre-formed G4 for sensing. Using the KANA aptamer as a model system, Yin et al. [22] investigated the roles of split fragments KANA-S1 and KANA-S2 in target detection by exploiting the affinity of ThT for G4 structures. Both KANA-S1 alone and the KANA-S1/S2 assembly were G-rich sequences capable of forming G4 conformations, which bound ThT and produced enhanced fluorescence (Figure 9c). Upon KANA binding, specific target–aptamer interactions disrupted the G4 structure, leading to ThT release and fluorescence quenching. The limit of detection for the KANA-S1/ThT system was 4.88 nM, while that for the KANA-S1/S2/ThT system was 4.53 nM. Under optimal experimental conditions, the constructed aptamer sensors showed recovery rates of 97.6% to 104.5% for the KANA-S1/ThT system and 98.4% to 105.9% for KANA-S1/S2/ThT system in spiked pretreated milk samples, indicating their applicability for KANA detection in milk (Table 2). These results confirmed the applicability and reliability of split aptamer-based fluorescence sensors for KANA detection in real food systems, particularly dairy products. This split aptamer G4-ThT platform faces similar matrix-related challenges. Free inorganic ions and other charged components in food matrices can influence G4 folding equilibria independent of target–aptamer recognition, potentially causing background fluorescence variation and reducing quantitative reliability in complex milk-derived samples.

Finally, the modularity of split aptamers allows their integration into sophisticated DNA nanostructures for multiplexed detection. DNA tweezers, a class of dynamic DNA nanomachines typically composed of three or four strands, can reversibly capture and release small molecules through conformational “opening” and “closing” motions [92]. Shang et al. [63] established a bifunctional switch-off DNA tweezer sensor to simultaneously detect enrofloxacin (ENR) and KANA in various food matrices (Figure 9d). Strand 1 (including KANA-S1) and Strand 2 (including KANA-S2), the two extended strands of the tweezer, were individually functionalized with split aptamers for ENR and KANA to act as recognition elements. Strands 3 and 4 were modified at their termini with a fluorescent reporter and a quenching group, respectively. Upon the addition of KANA, KANA-S1 and KANA-S2 recognition induced the formation of sandwich-type ternary complexes, driving the closure of the tweezer structure and resulting in a reduction in fluorescence intensity. In the absence of the target, the tweezers remained open, allowing the fluorescence signal to be sustained (Table 3). This multiplexed sensing strategy highlighted the potential of split aptamer-based nanodevices for the simultaneous monitoring of multiple antibiotic residues in milk and chicken. This bifunctional DNA tweezer enabled one-step dual detection of KANA and enrofloxacin in milk and chicken samples. However, the partially overlapping emission bands of FAM and ROX may introduce signal crosstalk during dual-target analysis. In addition, potential cross-response toward structurally related antibiotics should be systematically evaluated before application in complex food samples containing multiple antibiotic residues.

## 6. Conclusions and Future Perspectives

KANA contamination in food systems remains a critical global food safety concern, underscoring the urgent need for analytical methods that combine high sensitivity, rapid response, and operational simplicity. The development of KANA-specific aptamers has enabled the construction of fluorescent aptasensors as promising alternatives to conventional chromatographic and immunoassay techniques for food analysis. While several reviews have summarized KANA aptasensors, few have systematically addressed food-specific matrix interference mechanisms and rational split aptamer design principles. This review provides a systematic and critical analysis of the latest advances in fluorescent aptasensors for KANA detection, with a specific focus on their analytical performance and practical applicability in complex food matrices. We first map the evolutionary landscape of KANA-binding aptamers, from the first-generation KANA-1 to recently reported high-affinity variants, and critically address the enduring paradox that KANA-1 has been extensively deployed in sensor development despite the lack of confirmed solution-phase-specific binding activity. This discrepancy highlights critical limitations in early aptamer screening methodologies and indirect binding validation protocols. Nevertheless, the extensive body of work based on KANA-1 has yielded invaluable methodological insights into aptasensor design principles. Leveraging this clarified molecular foundation, we then systematically evaluate labeled and label-free sensing paradigms. Both approaches enable robust and sensitive KANA detection by translating aptamer–target recognition events into quantifiable fluorescence signals through nanomaterial-assisted transduction and nucleic acid amplification, exhibiting distinct advantages in adapting to the heterogeneous microenvironments of real food samples. In particular, split aptamer architectures effectively mitigate nonspecific folding, reduce synthetic complexity, and enhance assay robustness and kinetics, thereby improving detection reliability in heterogeneous food environments such as dairy systems.

Despite these advances, several challenges remain for practical implementation, including matrix variability across different food types, insufficiently validated sensitivity under complex food matrices, limited systematic assessment of batch-to-batch reproducibility in reported sensors, and the absence of standardized protocols for performance evaluation. Addressing these limitations will require the rational design of multifunctional nanocomposites to reduce nonspecific protein adsorption, the integration of efficient amplification strategies, and a deeper mechanistic understanding of aptamer–target interactions under realistic food conditions. Particular emphasis should be placed on structurally validated high-affinity aptamer variants in such mechanistic investigations. Future efforts should also focus on improving method robustness, simplifying sample pretreatment procedures, and enabling low-cost portable devices for rapid on-site detection across the entire food supply chain. In addition to physical and chemical strategies for sample pretreatment and interference suppression, the integration of data-driven signal processing provides a complementary approach for improving the robustness of fluorescence-based food sensing. Chemometric methods and machine learning algorithms can extract informative fluorescence features associated with target recognition from complex background variations, thereby reducing dependence on extensive sample pretreatment. For example, pattern recognition-based aptasensor arrays combined with classification algorithms have demonstrated the capability to distinguish target responses under different matrix conditions in food analysis. Machine learning-assisted fluorescent sensor arrays further exploit multidimensional optical fingerprints, including fluorescence intensity variations, ratiometric signals, and excitation–emission matrices, to improve analyte discrimination in complex sample environments. Moreover, smartphone-based fluorescence visual sensing platforms integrated with machine learning algorithms have expanded the feasibility of portable antibiotic detection by enabling automated signal interpretation and improved analytical reliability. Looking forward, the incorporation of machine learning into aptasensor design may facilitate real-time signal correction, multiplexed data deconvolution, and adaptive calibration across diverse food matrices. Nevertheless, practical implementation remains challenging due to the need for standardized data acquisition protocols, representative training datasets, model transferability across different food types, and interpretable lightweight algorithms suitable for portable devices. Addressing these challenges will be essential for translating laboratory-developed fluorescent aptasensors into reliable tools for real-world food safety monitoring.

Among chromatographic quantification methods, LC-MS/MS is widely accepted as a reference approach for antibiotic residue analysis because of its excellent specificity, accurate quantification capability, and capacity for simultaneous multi-analyte detection. Compared to LC-MS/MS, fluorescent aptasensors offer advantages in terms of portability, shorter analysis time, and lower instrumentation requirements. However, substantial challenges remain regarding standardized calibration procedures, matrix-independent quantitative accuracy, and long-term batch-to-batch reproducibility. These unresolved issues currently limit the regulatory acceptance and widespread application of fluorescent aptasensors in food safety monitoring. To facilitate the industrial translation and commercialization of aptamer-based sensing technologies, future efforts should focus on establishing standardized manufacturing processes, rigorous quality control criteria, systematic stability evaluation protocols, and comprehensive validation datasets across diverse food matrices. Such developments are essential to bridge the gap between laboratory-scale demonstrations and reliable field-deployable detection platforms.

Collectively, these developments are expected to advance fluorescent aptamer-based biosensors toward reliable, field-deployable platforms for routine food safety monitoring and the regulatory compliance of antibiotic residues. Amid the growing global crisis of antimicrobial resistance, these technologies will play an increasingly critical role in safeguarding public health.

## Figures and Tables

**Table 1:** Aptamer sequences of KANA and dissociation constants.

Aptamer	Aptamer Sequences (5′–3′)	Screening Method	K_d_ (Method)	Validation Method	Ref.
KANA-1	TGGGGGTTGAGGCTAAGCCGA	Affinity Chromatography SELEX	78.8 nM	FAM-labeled aptamer binding to KANA-coated beads	[36]
KANA-2.1	AGCCCGGTATTGAGGTCGATCTCTTATCCTATGGCTTGTCCCCCATGGCTCGGTTATATCC	Capture-SELEX	3900 nM	Ligand-induced release	[39]
KANA-2.2	CAGGGCGGTATGAGGCTCGATCAAGGTCGGAGCGAGAATTTTTTCGCGGAGTCGGCTGGATC	Capture-SELEX	5100 nM	Ligand-induced release	[39]
KANA-3	CGGAAGCGCGCCACCCCATCGGCGGGCGCGAAGCTTGCG	NOS-immobilized SELEX	92.3 nM	ELONA	[40]
KANA-4	GAGGGCCTGAAACTTGCTGGAACGGTTTAA	Capture-SELEX	320 nM	ITC	[37]
KANA-5	GACGACGCAGTCGGCTTTTGCGGGAAAAAGGTCGGAGTCGTC	Capture-SELEX	51 ± 5 nM	ITC	[41]

**Table 2:** Comparison of various fluorescent aptasensor methods for KANA detection.

Aptamer	Strategy	LOD (nM)	Linear Range (nM)	Sample	Assay Conditions	Sample Pretreatment	Ref.
KANA-1	AuNPs/FAM	0.3	0.8–350	Milk	PB, pH 8.2, 4 °C, 120 min	SPE column purification after protein precipitation	[42]
KANA-1	AuNPs/FAM	0.0001	0.0001–100	River water, Lake water, Milk	PBS, pH 8.0, 4 °C, 45 min	Milk: acetonitrile extraction, rotary evaporation; water samples: sedimentation, supernatant collection	[43]
KANA-1	AuNPs/Cy3	71.53	200–10,000	Milk	PBS, pH 7.4, 37 °C, 60 min	Acetic acid precipitation, centrifugation, filtration	[44]
KANA-1	AuNPs/FAM	23.6	50–800	Milk	PBS, pH 7.4, 37 °C, 60 min	Acetic acid precipitation, centrifugation, filtration	[45]
KANA-1	UCNPs/graphene	0.018	0.03–3	Human serum	Tris-HCl, pH 8.0, 4 °C, 210 min	100-fold dilution	[24]
KANA-1	CNTs/FAM	0.4	1–50	Milk	Tris-HCl, pH 8.0, RT, 45 min	Acetic acid precipitation, centrifugation, filtration, neutralization	[46]
KANA-1	GO/DNase I amplification	5498.4	20,640–103,199	Milk	Tris-HCl, pH 8.0, RT, 60 min	Ethyl acetate extraction for fat removal	[47]
KANA-1	DNAzyme/magnetic beads	0.805	2.06–4,127,967	Milk	PBS, pH 7.4, 37 °C, 140 min	Centrifugation, 100-fold dilution	[48]
KANA-1	Exo I/2-AP/μPAD	1.26 × 10^−5^	0.0001–100	Milk, Honey	Tris-HCl, pH 6.8, 37 °C, 20 min	Milk: Acetic acid precipitation, centrifugation, filtration; honey: PBS dilution, ultrasonication, centrifugation, filtration	[49]
KANA-1	Exo III/2D-N@CNFs	0.0158	0.05–100	Fish	Tris-HCl, pH 8.0, 37 °C, 120 min	Homogenization, centrifugation	[50]
KANA-1	CHA/FAM-BHQ1	54	54–900	Milk	Tris-HCl, pH 7.5, 37 °C, 60 min	Acetic acid precipitation, centrifugation, filtration	[51]
KANA-1	L-CHA/DNA tetrahedron	39.22	2064–10,319,917	Milk	PBS, 37 °C, 60 min	Centrifugation, 5-fold PBS dilution	[52]
KANA-1	G4/TO	59	100–2000	Milk	Tris-HCl, pH 7.2, 25 °C, 10 min	Acetonitrile and TCA precipitation, centrifugation	[21]
KANA-1	G4/ThT	0.3	1–300,000	Milk	Tris-HCl, pH 7.0, 30 °C, 20 min	Acetonitrile precipitation, centrifugation, filtration	[53]
KANA-1	G4/ThT	0.37	0.7–10	Milk	Tris-HCl, pH 7.5, 37 °C, 30 min	NaOH blending, ultrasonication, acetic acid to pH 4.6, centrifugation, neutralization, 10-fold dilution	[54]
KANA-1	G4/NMM	0.5	0.5–100	Milk	Tris-HCl, pH 7.5, RT	NaOH blending, ultrasonication, acetic acid to pH 4.6, centrifugation, neutralization, 10-fold dilution	[55]
KANA-1	G4/NMM	0.60	1–400	Milk	TNaK, pH 7.5, 37 °C, 30 min	Acetic acid precipitation, centrifugation, filtration	[56]
KANA-1	HCR/SG I	9.29 × 10^−4^	2.06–206,398	Milk, Fish, Pork	PBS, pH 7.4, 25 °C, 60 min	Milk: TCA precipitation, centrifugation, filtration; fish/pork: homogenization, centrifugation, supernatant collected	[19]
KANA-1	CDs/AuNPs IFE	18	40–240	Milk	Tris-HCl, pH 8.0, 30 °C, 60 min	Acetonitrile precipitation, centrifugation, filtration	[23]
KANA-1	GOQDs/AuNPs IFE	3.6	5–600	Milk, Honey, Human serum	PBS, pH 7.4, RT, 130 min	Milk: Acetonitrile precipitation, centrifugation, filtration; honey: PBS dilution, sonication, centrifugation, filtration; serum: 1000-fold dilution	[57]
KANA-1	AgNCs	0.18	0.5–4	Milk	TE, pH 6.0, RT, 2 min	Methanol precipitation, centrifugation, filtration, nitrogen drying, reconstitution	[58]
KANA-1	HCA/AgNCs	1.35 × 10^−5^	0.02064–20,640	Milk	PBS, pH 7.4, 37 °C, 110 min	PBS dissolution, centrifugation	[26]
KANA-1	CuNCs@SiO_2_/DNA-AgNCs/Au@Pd nanoparticles	7.3	50–1000	Milk, Honey, Beef	TE, pH 7.5, RT, 125 min	Homogenization, centrifugation, filtration	[27]
KANA-1	AgNCs/AuNPs SPEET	1.0	5–50	Milk	PBS, pH 7.0, RT, 25 min	Methanol precipitation, centrifugation, filtration, nitrogen drying, reconstitution	[59]
KANA-1	DNA-AgNCs/AuNPs FRET	22.6	100–1100	Milk	TE, pH 7.8–8.2, RT, 120 min	Methanol precipitation, centrifugation, filtration, nitrogen drying, reconstitution	[60]
KANA-1	AuNCs/MnO_2_ nanosheets/3WJ-HCR amplification	1.2 × 10^−3^	0.002–5	Milk	HEPES, pH 7.4, 37 °C, >240 min	TCA and methanol precipitation, centrifugation, filtration	[25]
KANA-S1/S2	CuAAC	0.026	0.04–20	Human serum	PBS, pH 7.4, RT, 120 min	Direct detection	[61]
KANA-S1/S2	DNA-AgNCs/Exo I	1.07	5–1000	Oyster meat, Seawater, Lake water, Milk	TE, pH 6.75, 37 °C, 35 min	Oyster: homogenization, centrifugation, filtration; milk: acetonitrile precipitation, centrifugation, filtration; water: 0.22 μm filtration	[62]
KANA-S1/S2	ThT/G4	4.53	10–7000	Milk	Tris-HCl, pH 5.0, 35 °C, 10 min	TCA precipitation, centrifugation, filtration, 100-fold dilution	[22]
KANA-S1/S2	DNA tweezer/FAM	743.03	1032–2,063,983	Milk, Chicken	PBS, pH 7.5, 37 °C, 90 min	Milk: pH 4.4, centrifugation to remove fat, filtration; chicken: TCA/acetonitrile extraction, centrifugation, pH adjustment, filtration	[63]
KANA-3	RGO/FAM	0.001	0.001–0.02	Blood serum, Milk	PBS, pH 8.5, 23 °C, 55 min	Serum: direct detection; milk: acetonitrile precipitation, centrifugation, filtration;	[40]
KANA-4	pH-directed/ThT	100	\	Human serum, Lake water	MES, pH 6.0, 25 °C	Lake water: buffered to pH 7; Serum: 10% dilution	[37]
KANA-5	Strand-displacement/FAM	900	\	Human serum	HEPES, pH 7.5, 22 °C	Direct detection	[41]

**Table 3:** Split aptamer sequences for KANA.

Aptamer	Aptamer Sequences (5′–3′)	Parent Aptamer	Screening Method	K_d_ (Method)	Validation Method	Ref.
KANA-S1	TGGGGGTTGAG	KANA-1: TGGGGGTTGAGGCTAAGCCGA	\	\	\	[61]
KANA-S2	GCTAAGCCGA		\	\	\	[61]

## Data Availability

No new data were created or analyzed in this study. Data sharing is not applicable to this article.

## Abbreviations

KANA	Kanamycin
MRLs	Maximum residue limits
GC	Gas chromatography
HPLC	High-performance liquid chromatography
LC-MS	Liquid chromatography–mass spectrometry
SELEX	Systematic Evolution of Ligands by Exponential Enrichment
SG I	SYBR Green I
dsDNA	Double-stranded DNA
TO	Thiazole orange
ThT	Thioflavin T
G4	G-quadruplex
CDs	Carbon dots
UCNPs	Upconversion nanoparticles
AuNCs	Gold nanoclusters
AgNCs	Silver nanoclusters
CuNCs	Copper nanoclusters
RCA	Rolling circle amplification
SDA	Strand displacement amplification
HCR	Hybridization chain reaction
CHA	Catalytic hairpin assembly
K_d_	Dissociation constant
T_m_	Melting temperature
ITC	Isothermal titration calorimetry
NOS	Nitrocellulose oligosorbent
ssDNA	Single-stranded DNA
ELONA	Enzyme-linked oligonucleotide assay
NMR	Nuclear magnetic resonance
CuAAC	Cu(I)-catalyzed azide–alkyne cycloaddition
AuNPs	Gold nanoparticles
FRET	Förster resonance energy transfer
SNA	Spherical nucleic acid
CNTs	Graphene and carbon nanotubes
UC-FRET	Upconversion fluorescence resonance energy transfer
GO	Graphene oxide
SET	Surface energy transfer
LOD	Limit of detection
CESA	Cyclic enzyme signal amplification
cDNA	Complementary DNA
MBs	Magnetic beads
2-AP	2-Aminopurine
μPAD	Microfluidic paper-based analytical device
Exo III	Exonuclease III
2D-N@CNFs	Two-dimensional N-doped carbon nanoflares
RET	Resonance energy transfer
L-CHA	Local catalytic hairpin assembly
IFE	Inner filter effect
GOQDs	Graphene oxide quantum dots
TOB	Tobramycin
CuNCs@SiO_2_	Silica-encapsulated copper nanoclusters
DNA-AgNCs	DNA-templated silver nanoclusters
SPEET	Surface plasmon-enhanced energy transfer
SPR	Surface plasmon resonance
3WJ-HCR	Three-way junction hybridization chain reaction
ALP	Alkaline phosphatase
AA	Ascorbic acid
ENR	Enrofloxacin
